# CD4+ αβ T cell infiltration into the leptomeninges of lumbar dorsal roots contributes to the transition from acute to chronic mechanical allodynia after adult rat tibial nerve injuries

**DOI:** 10.1186/s12974-018-1115-7

**Published:** 2018-03-15

**Authors:** Bin Du, You-Quan Ding, Xia Xiao, Hong-Yi Ren, Bing-Yin Su, Jian-Guo Qi

**Affiliations:** 1 Department of Histology, Embryology and Neurobiology, West China School of Preclinical and Forensic Medicine, Sichuan University, No 17, Section 3, South Ren-min road, Chengdu, Sichuan 610041 China; 2 Sichuan Province Development and Regeneration Key Laboratory, Chengdu Medical College, Chengdu, Sichuan 610500 China

**Keywords:** Peripheral nerve injury, Neuropathic pain, Chronic pain, Mechanical allodynia, CD4+ αβ T cell, Dorsal root, Leptomeninges, Lymph node, Glia, PKCγ^+^ excitatory interneuron

## Abstract

**Background:**

Antigen-specific and MHCII-restricted CD4+ αβ T cells have been shown or suggested to play an important role in the transition from acute to chronic mechanical allodynia after peripheral nerve injuries. However, it is still largely unknown where these T cells infiltrate along the somatosensory pathways transmitting mechanical allodynia to initiate the development of chronic mechanical allodynia after nerve injuries. Therefore, the purpose of this study was to ascertain the definite neuroimmune interface for these T cells to initiate the development of chronic mechanical allodynia after peripheral nerve injuries.

**Methods:**

First, we utilized both chromogenic and fluorescent immunohistochemistry (IHC) to map αβ T cells along the somatosensory pathways for the transmission of mechanical allodynia after modified spared nerve injuries (mSNIs), i.e., tibial nerve injuries, in adult male Sprague-Dawley rats. We further characterized the molecular identity of these αβ T cells selectively infiltrating into the leptomeninges of L4 dorsal roots (DRs). Second, we identified the specific origins in lumbar lymph nodes (LLNs) for CD4+ αβ T cells selectively present in the leptomeninges of L4 DRs by two experiments: (1) chromogenic IHC in these lymph nodes for CD4+ αβ T cell responses after mSNIs and (2) fluorescent IHC for temporal dynamics of CD4+ αβ T cell infiltration into the L4 DR leptomeninges after mSNIs in prior lymphadenectomized or sham-operated animals to LLNs. Finally, following mSNIs, we evaluated the effects of region-specific targeting of these T cells through prior lymphadenectomy to LLNs and chronic intrathecal application of the suppressive anti-αβTCR antibodies on the development of mechanical allodynia by von Frey hair test and spinal glial or neuronal activation by fluorescent IHC.

**Results:**

Our results showed that during the sub-acute phase after mSNIs, αβ T cells selectively infiltrate into the leptomeninges of the lumbar DRs along the somatosensory pathways responsible for transmitting mechanical allodynia. Almost all these αβ T cells are CD4 positive. Moreover, the temporal dynamics of CD4+ αβ T cell infiltration into the lumbar DR leptomeninges are specifically determined by LLNs after mSNIs. Prior lymphadenectomy to LLNs specifically reduces the development of mSNI-induced chronic mechanical allodynia. More importantly, intrathecal application of the suppressive anti-αβTCR antibodies reduces the development of mSNI-induced chronic mechanical allodynia. In addition, prior lymphadenectomy to LLNs attenuates mSNI-induced spinal activation of glial cells and PKCγ^+^ excitatory interneurons.

**Conclusions:**

The noteworthy results here provide the first evidence that CD4+ αβ T cells selectively infiltrate into the DR leptomeninges of the somatosensory pathways transmitting mechanical allodynia and contribute to the transition from acute to chronic mechanical allodynia after peripheral nerve injuries.

**Electronic supplementary material:**

The online version of this article (10.1186/s12974-018-1115-7) contains supplementary material, which is available to authorized users.

## Introduction

For susceptible animals and humans, neuropathic pain following peripheral nerve injuries persists long after the initial damage has subsided [1, 2]. This chronic debilitating disease typically manifests as an increased sensitivity to mechanical or thermal stimuli, is resistant to conservative medical management, and significantly decreases the quality of life [1, 2]. Mechanical allodynia, a painful response to innocuous mechanical stimuli, such as gentle touch, is one of the most problematic symptoms for these patients [3–6]. Upon peripheral nerve injuries, the nearby spared primary sensory neurons (PSNs) are sensitized to a dysregulated molecular and functional state [7]. The resulting central sensitizations within the spinal cord dorsal horn (SC-DH) (including synapse facilitation and disinhibition) open or overcome the inhibitory gate for low-threshold Aβ mechanoreceptor inputs, thus allowing innocuous mechanical stimuli to directly activate the normally silent and polysynaptic neural circuit for mechanical pain [3–7]. For persistent neuronal sensitizations and chronic mechanical allodynia, the long-lasting reciprocal signaling between glial cells and neurons within peripheral nerves and SC-DHs has been now widely recognized as the key process [8–10].

A growing body of evidence has demonstrated that T cells but not B cells are critical for the development of mechanical allodynia after peripheral nerve injuries [11–17]. Further studies showed that in contrast to CD8+ αβ T cells, B cells, NK cells, and macrophages, CD4+ αβ T cells were selectively activated by dendritic cells and polarized to IFNγ-positive, inflammatory Th1 cells in peripheral lymphoid organs, such as the spleen, after peripheral nerve injuries [12, 18]. These antigen-specific, MHCII-restricted CD4+ αβ T cells were shown to contribute to the transition of acute mechanical allodynia to a chronic state after nerve injuries and persistent glial activation within the spinal cord (SC) [11, 12, 15, 18–20]. However, it is still unknown where CD4+ αβ T cells infiltrate along the somatosensory pathways transmitting mechanical allodynia to initiate the development of chronic mechanical allodynia. Although αβ T cells have been conclusively found to infiltrate into the injured nerves [11, 21–25] and might also enter into the cell-body-rich areas of dorsal root ganglia (DRGs) after nerve injuries [21, 22, 24–28], it remains uncertain whether these T cells are CD4 positive and contribute to the transition from acute to chronic mechanical allodynia. The SC-DH has been regarded as an important neuroimmune interface for T cells to act on the somatosensory pathways for the transmission of mechanical allodynia to initiate the development of chronic mechanical allodynia [12, 13, 16, 18, 27, 29–31], but accumulating studies doubted the presence of CD4+ αβ T cells, even T cells, in the SC-DHs after nerve injuries [16, 18, 21, 22, 24, 32–35].

**Fig. 1:**
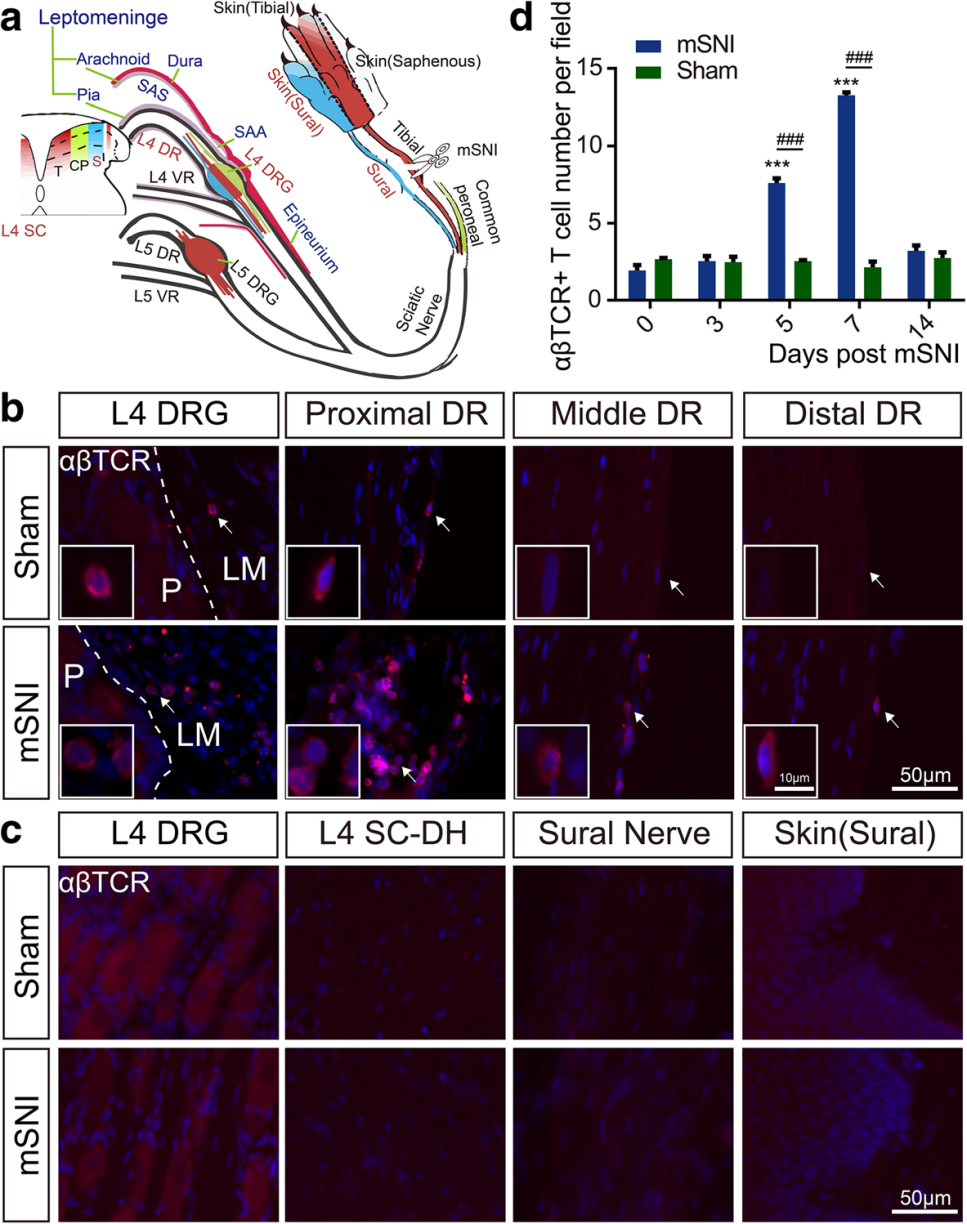
Mapping of αβ T cells along the somatosensory pathways after adult rat mSNIs. **a** Schematic illustration of the target tissues (in red font), which were mapped for αβ T cells along the somatosensory pathways for the transmission of mechanical allodynia on the glabrous sural skin territories after mSNIs or sham surgeries (*n* = 5/group). The L4 SC-DH is somatotopically divided along the mediolateral axis into the injured tibial innervation territories (~ medial 45%), the intact common peroneal innervation territories (~ central 1/4) and the intact sural innervation territories (~ lateral 1/3). The proximal and distal stumps of the injured tibial nerves and the hindpaw glabrous skins innervated by the tibial nerves were also examined. **b** The infiltration of αβ T cells across the whole course of the L4 DR leptomeninges 7 days after mSNIs and sham operations. **c** The infiltration of αβ T cells in the cell-body-rich areas of L4 DRGs, L4 SC-DHs, the sural nerves, and the hindpaw glabrous sural skins 7 days after mSNIs and sham operations. **d** The temporal dynamics of αβ T cell infiltration into the leptomeninges covering the proximal L4 DRs at the DR portions of the SAAs after mSNIs and sham operations. ^***^P < 0.001; after versus before mSNIs. ^###^*P* < 0.001; mSNI versus sham operation. CP common peroneal, DR dorsal root, DRG dorsal root ganglion, LM leptomeninge, mSNI modified spared nerve injury, P parenchyma, SAA subarachnoid angle, S sural, SAS subarachnoid space, SC spinal cord, SC-DH spinal cord dorsal horn, T tibial, VR ventral root

Recently, the cerebrospinal meninges have been shown as the critical and multifaceted neuroimmune interface for beneficial or detrimental T cell cross-talks with the central nervous system (CNS) during homeostasis and diseases [36]. These meninges harbor MHCII-expressing cells, such as macrophages and dendritic cells (DCs), which line on the leptomeningeal (including pia and arachnoid mater) surfaces adjacent to the cerebrospinal fluid (CSF) compartments for antigen processing [36]. Lymphatic vessels in the dura mater and epidural tissues are responsible for antigen drainage from the CSF to local lymph nodes, such as the deep cervical lymph nodes (CLNs) [36]. Moreover, the cerebrospinal leptomeninges are rich in blood microvessels and thus permissive to circulating T and B lymphocytes [36]. A series of seminal studies [26–28] suggested that after peripheral nerve injuries, αβ T cells robustly infiltrate into the leptomeninges of the subarachnoid angles (SAAs) at the transitional zone between the lumbar dorsal roots (DRs) and DRGs (Figs. 1a and 3b) [37, 38]. However, the molecular identity of these αβ T cells and their roles in the development of chronic mechanical allodynia remain exclusively unknown.

Meninges of DRs are likely to be functionally similar, if not identical, to cerebrospinal meninges for T cell interactions with the nervous systems, since these meninges are presumably regarded as a continuation of spinal meninges and potentially have similar immunological elements [37–42]. Therefore, in the present study, we mapped the presence of αβ T cells along the somatosensory pathways transmitting mechanical allodynia on the glabrous sural skin territories after tibial nerve injuries, characterized the molecular identity of αβ T cells in lumbar DR leptomeninges, and investigated whether CD4+ αβ T cells infiltrating into the leptomeninges of adult rat lumbar DRs initiate the development of chronic mechanical allodynia. Our noteworthy results here provide the first evidence for the DR leptomeninges as the new and definite neuroimmune interface for CD4+ αβ T cells to initiate the development of chronic mechanical allodynia after nerve injuries.

## Materials and methods

### Animals and the modified spared nerve injury

Twelve-week old, adult male Sprague-Dawley (SD) rats, weighing 300–350 g at the beginning of the study, were obtained from the Laboratory Animal Center of Sichuan University and used in the present study. The animals were housed with free access to standard chow and water in a room with an ambient temperature of 22 ± 1 °C and a 12:12 h light/dark cycle. All animal manipulations were performed with the approval of the Animal Care and Ethics Committee of Sichuan University and in strict accordance with the National Institutes of Health Guide for the Care and Use of Laboratory Animals (publication no. 85–23, revised 1985).

Adult rats were subjected to the modified spared nerve injuries (mSNIs), i.e., tibial nerve injuries (see Additional file 1: Figure S1A) [43, 44]. In brief, the sciatic nerves from the right hindlimbs were exposed around their trifurcations, and the identified tibial nerves were gently freed of the surrounding connective tissues. Then, the tibial nerves were tightly ligated and transected just distal to the trifurcations of the sciatic nerves. Distal to the injury sites, about 5-mm long segments of tibial nerves were removed to prevent spontaneous regeneration. Any potential damage to the common peroneal and sural nerves was avoided. Sham surgery for the mSNI just involved the exposure of the tibial nerve.

Compared with the original SNI pain model (transecting both the tibial and common peroneal nerve branches), the mSNI pain model is simpler in model construction. Moreover, this model is more convenient in behavioral tests due to the elimination of the eversion of the hindpaw plantar surfaces, which is frequently seen in the original SNI model. The mSNI model in adult male SD rats showed significant and persistent behavioral signs of mechanical allodynia on the glabrous sural nerve skin territories of the ipsilateral hindpaws (see Additional file 1: Figure S1B, C). To provide the rationality of the mSNI neuropathic pain model for our present study, our unpublished observations in animals with prior splenectomy indicated that antigen-specific and MHC II-restricted CD4+ αβ T cells also contribute to the development of chronic mechanical allodynia after adult rat mSNIs.

### Immunohistochemistry for αβ T cells along the somatosensory pathways

Seven days after mSNIs or sham surgeries (*n* = 5 animals per group), we utilized both chromogenic and fluorescent immunohistochemistry (IHC) to map the infiltration of αβ T cells into the tissues along the somatosensory pathways. After paraformaldehyde fixative-mediated transcardial perfusion fixation, adult rat tissues from the skin up to the SC along the somatosensory pathways (Fig. 1a) were carefully harvested and serially sectioned on a cryostat into 10-μm thick, slide-mounted thin sections. In brief, the glabrous skins, specifically the tibial and sural skin areas as well as the adjacent α and β footpads, were removed from the ipsilateral hindpaws to the mSNI or sham surgery and sliced perpendicularly to the skin surface. The proximal and distal stumps of directly injured tibial nerves and the nearby intact sural nerves were harvested and longitudinally sectioned. The L4 DRGs with the attaching proximal portions of L4 DRs, the middle portions of L4 DRs floating in the CSF, and the distal portions of L4 DRs attached to L4 SCs were gently isolated and longitudinally sectioned. Moreover, transverse sections of L4 SC segments were prepared. Representative sections across the whole volume of the target tissues (the sampling ratio as 1:3) were processed for IHC as described previously [45, 46]. Coherent parallel processing, appropriate controls, and blind experimental design were used to minimize systematic biases or errors, allowing relatively feasible intergroup comparisons in these and other IHC experiments.

Sections of these tissues along the somatosensory pathways were first processed for αβTCR chromogenic IHC as described previously [18]. In brief, sections were incubated overnight at 4 °C with mouse anti-αβTCR monoclonal primary antibodies (PAbs) (1:1000, clone R73, Bio-Rad Laboratories Inc., MCA53GA; Hercules, CA, USA). Then, the thoroughly rinsed sections were incubated with biotin-conjugated donkey anti-mouse secondary antibodies (SAbs) (1:500, Proteintech, SA00004-9; Wuhan, Hubei, China) for 2 h at room temperature (RT) and finally with HRP-conjugated streptavidin (1:500, Beyotime, A0303; Shanghai, China) for 1 h at RT. The sections were developed with DAB substrate kit (ZSGB-BIO, ZLI-9017; Beijing, China) for immunoreactive signals and then counterstained with Mayer’s hematoxylin (Abcam, ab220365; Cambridge, UK). The potential αβ T cells in these sections were imaged across the whole tissue areas with a × 20 (NA = 0.75) or × 40 (NA = 0.75) Olympus UPLSAPO objective, which was equipped on a fully motorized, Olympus IX-83 widefield (WF) epifluorescence microscope with a DP80 CCD camera under the color mode controlled by Olympus cellSens Dimension software (Olympus; Tokyo, Japan).

Another set of sections from these tissues was processed for αβTCR fluorescent IHC as described previously [45]. In brief, sections were incubated first overnight at 4 °C with PAbs against αβTCR (1:500). Then, the thoroughly rinsed sections were incubated 2 h at RT with highly cross-absorbed donkey anti-mouse F(ab’)_2_ IgG SAbs conjugated to DyLight 594 (1:1000, Abcam, ab98768). Our pilot studies showed that the employment of fragment SAbs significantly reduced the non-specific staining via the interaction of IgG Fc regions with Fc receptors in the tissues. The cell nuclei were stained with DAPI before mounting. The potential αβ T cells in these sections across the whole tissue areas were imaged with a × 20 (NA = 0.75) or × 40 (NA = 0.75) Olympus UPLSAPO objective, which was equipped on a fully motorized, Olympus IX-83 WF epifluorescence microscope with a DP80 CCD camera under the monochromatic mode controlled by Olympus cellSens Dimension software. Representative images of almost the same anatomical localization in the matched sections were blindly selected for the assembly of the figures of this publication.

### Temporal dynamics and molecular identity for αβ T cells infiltrating into the lumbar dorsal root leptomeninges

For mSNIs or sham-operated animals (*n* = 5 animals per group at each designated time point), slide-mounted sections (10-μm thick) of L4 DRGs with the attaching proximal DRs were prepared and processed for αβTCR single fluorescent immunolabeling (1:500) before and at serial designated time points after mSNIs. We used the proximal L4 DRs to quantitatively profile the temporal dynamics of αβ T cell infiltration into the leptomeninges of L4 DRs for two reasons. First, the leptomeninges covering the middle or distal L4 DRs are just the pia mater and vulnerable to the potential surgical damage during tissue harvesting (Figs. 1a and 3b) [37]. Second, the proximal L4 DRs were covered by both the pia and arachnoid mater at the DR portions of the SAAs (Figs. 1a and 3b) [37], and our pilot studies showed that the leptomeninges covering the proximal L4 DRs have the highest number of αβ T cells across the whole L4 DRs after mSNIs. The αβ T cells in the leptomeninges covering the proximal L4 DRs at the DR portions of the SAAs (Figs. 1a and 3b) were determined with a × 20 (NA = 0.75) UPLSAPO objective (at least three fields/ section) or a × 40 (NA = 0.75) Olympus UPLSAPO objective. Images were captured from almost the same fields in the matched sections from mSNIs or sham-operated animals and quantitatively profiled with the cell counter plug-in of Fiji software for temporal dynamics of αβ T cell infiltration into the lumbar dorsal root leptomeninges after mSNIs. For lymphadenectomized or sham-operated animals to the LLNs (*n* = 5 animals per group at each designated time point), the same methods of staining, imaging, and quantification were used for temporal dynamics of αβ T cell infiltration into the lumbar dorsal root leptomeninges after mSNIs. Moreover, 7 days after mSNIs, the numbers of αβ T cells infiltrating into the proximal or distal portions of the injured tibial nerves were similarly assessed in lymphadenectomized or sham-operated animals to the LLNs and the sciatic or popliteal lymph nodes (*n* = 5 animals per group for each group of lymph nodes).

Five or 7 days after mSNIs (*n* = 5 animals at each designated time point), a set of sections for all the portions of the lumbar DRs was prepared and processed for molecular characterization of αβ T cells infiltrating into the lumbar DR leptomeninges. We utilized sequential double fluorescent immunolabeling with CD4/CD8 and αβTCR. In brief, sections were incubated first overnight at 4 °C with the following PAbs: mouse monoclonal anti-CD4 (1:1000, clone W3/25, Bio-Rad Laboratories Inc., MCA55GA) or mouse monoclonal anti-CD8 (1:1000, clone OX-8, Bio-Rad Laboratories Inc., MCA48GA). Then, the thoroughly rinsed sections were incubated 2 h at RT with highly cross-absorbed donkey anti-mouse F(ab’)_2_ IgG SAbs conjugated to DyLight 594 (1:1000, Abcam, ab98768). Following extensive washing and enough blocking, the sections were further incubated overnight at 4 °C with biotin-conjugated, mouse anti-αβTCR monoclonal PAbs (1:500, clone R73, Invitrogen, MA5-17542; Eugene, OR, USA) and 2 h at RT with streptavidin conjugated to Alexa Fluor 488 (1:500, Abcam, ab150116). The cell nuclei were stained with DAPI before mounting. The potential CD4+ αβ or CD8+ αβ T cells in these sections across the whole tissue areas were imaged with a × 20 (NA = 0.75) or × 40 (NA = 0.75) Olympus UPLSAPO objective on an Olympus FV1000 confocal microscope. Representative images for each portion of the lumbar DRs were blindly selected for the assembly of the figures of this publication. Images from the leptomeninges covering the proximal L4 DRs at the DR portions of the SAAs were quantitatively profiled with the cell counter plug-in of Fiji software for the percentage ratio of CD4+ or CD8+ cells in the αβ T cell populations.

### Immunohistochemistry for CD4+ αβ T cells in the lumbar lymph nodes

Seven days after mSNIs or sham surgeries, the lumbar lymph nodes (LLNs) of adult rats (*n* = 5 animals per group) were removed to determine reactive hypertrophy and relative weights of those pooled peripheral lymphoid organs to their corresponding body weights as described previously [18]. At the same time points, the LLNs of adult rats (*n* = 5 animals per group) were longitudinally sectioned on a cryostat in 10-μm thickness and processed for chromogenic IHC as described previously [18]. In brief, slide-mounted frozen sections of LLNs were incubated overnight at 4 °C with the following PAbs: mouse monoclonal anti-αβTCR (1:1000), mouse monoclonal anti-CD4 (1:1000), and rabbit polyclonal anti-IFNγ (1:100, BioLegend, 507801; San Diego, CA, USA). The positive cells in the paracortical zones (PCZs) of these lymph nodes were imaged with a × 20 (NA = 0.75) Olympus UPLSAPO objective (at least three fields/section, at least eight sections/sample).

### Prior lymphadenectomy to the draining local lymph nodes along the somatosensory pathways

Seven days before mSNIs, adult rats were subjected to prior lymphadenectomy or sham surgeries. Four groups of local lymph nodes (lumbar, cervical, right popliteal, and right sciatic lymph nodes) were surgically removed respectively (Fig. 5a) [47]. The sham surgeries just involved the exposure of the corresponding group of local lymph nodes, without the removal of the lymph nodes. Antibiotics and analgesics were used during the perioperative and recovery periods. At the end of the experiments, animals were carefully examined for lymph nodes possibly left behind or regenerated.

For lymphadenectomy to LLNs and CLNs, we adopted the surgical procedures used in mice (see Additional file 8: Figure S8A, B) [48, 49]. For the LLNs (also called iliac and caudal nodes), a median incision was made into the skin and peritoneum of the lower abdomen, and the intestines were then retracted to gain access to the rat LLNs, which lie singly or in pairs along each side of the distal abdominal aorta where the aorta bifurcates into the iliac arteries [47, 48]. All the LLNs were removed. Then, the intestines were replaced, and the incisions were closed in layers. For the CLNs [47–49], a median incision was made into the skin overlying the submandibular glands. With the help of a surgical microscope, the eight superficial CLNs (also called submandibular, facial, and jugular lymph nodes) were identified around the surfaces of the submandibular glands and carefully removed with a pair of microforceps. Then, the submandibular glands and sternocleidomastoid muscles were extracted to expose the deep CLNs (also called internal jugular lymph nodes), which lie paratracheally. The two deep cervical lymph nodes were then delicately excised. Following the glands and muscles replaced, the incisions were closed in layers.

For the sciatic lymph nodes (SLNs) of right hindlimbs, we developed the following surgical procedure (see Additional file 8: Figure S8C) [28, 47]. A longitudinal incision was made right lateral to the sciatic notch. After the separation of the overlying muscles, the SLN was exposed and removed. Following the muscles replaced, the incisions were closed in layers. For the popliteal lymph nodes (PLNs) of right hindlimbs, we developed the following surgical procedure (see Additional file 8: Figure S8D) [21, 22, 47]. An arch incision was made into the skin from the trochanter tertius and the fibular head. After gentle separation of the anterior and posterior head of the biceps femoris, the PLN was identified to accompany the popliteal vein within the popliteal fossa. After careful isolation from the surrounding adipose tissues, muscles, and nerves, the lymph node was removed and the incisions were closed in layers.

### Chronic lumbar intrathecal application for targeted suppression of CD4+ αβ T cells in lumbar dorsal root leptomeninges

Seven days before mSNIs, adult rats were subjected to lumbar intrathecal catheterization (Fig. 6a). With some modifications of previously reported protocols [50, 51], we established the following protocol for lumbar intrathecal catheterization in adult rats. In brief, an intrathecal delivery system (Fig. 6a) was firstly self-assembled for long-term bolus injection. This dosing system included a 16-cm-long piece of PE-10 tubing catheter (RWD Life Science 62324, ID 0.28 mm × OD 0.61 mm), which was marked 1.5 cm from its one end with an ethanol-resistant marker pen. The other end of the catheter was externalized with an infusion guide cannula (RWD Life Science 62001, ID 0.45 mm × OD 0.64 mm) through a 2-cm-long piece of PE-50 tubing adapter (RWD Life Science 62327, ID 0.58 mm × OD 0.97 mm). Before this externalization process, the 3-cm-long, straight tube below the plastic pedestal of the infusion guide cannula was rendered to an L-shape tube. The infusion guide cannula was further fixed to a 6-mm-long barrel with the flanged base of the 1-mL syringe via a doughnut-like plug, which was cut from the rubber plug on the internal plunger of the same 1-mL syringe. A 3-mm hole was drilled in the center of each of the flange for subcutaneously mounting the externalization device on the muscle.

Then, the self-made intrathecal bolus delivery system was implanted in vivo into the lumbar arachnoid space and anchored to the back musculature of the mid-thoracic region (Fig. 6a). In brief, two longitudinal skin incisions were made on the back at the mid-thoracic region caudal to the caudal angles of the scapulae and the lumbrosacral region. With the help of a long and straight 16-gauge needle, the catheter was tunneled subcutaneously from the rostral skin incision to the caudal skin incision. The externalization device was subcutaneously sutured to the back musculature underlying the rostral skin incision through the holes in the flanges of the syringe barrel. Addition suture was made to the proximal catheter to anchor it to the underlying muscles, the rostral skin incision was closed, and the infusion guide cannula was capped. Then, at the region of the caudal skin incision, a suture was made to the distal catheter to anchor it to the fascia and muscles between the spinal processes of L4 and L5 vertebrae. The distal catheter was looped left to the vertebral column and fixed to the underlying flank musculature. The spinal processes of L6 and S1 vertebra were exposed, and the S1 spinal process was removed. With the help of a long and beveled 16-gauge needle, the distal catheter was tunneled through the paravertebral muscle to the dorsal surface of the S1 vertebral lamina. A 23-gauge needle was rostrally inserted at an angle of 45° into the identified depression between L6 and S1 vertebrae, and the distal catheter was carefully inserted through the punctured hole into the lumbar subarachnoid space bit by bit in the rostral direction. The pre-made indelible mark was used as a reference for the insertion length of the catheter into the subarachnoid space and therefore placed the catheter tip intrathecally at the upper margin of L5 vertebrae. The catheter was covered with a piece of sterile gelfoam, and the muscle and skin incision were closed layer by layer. The animal was allowed to recover before the returning to its housing cage and carefully cared during the post-operative course. The potential influences of lumbar intrathecal catheterization on behavioral performances were excluded before mSNIs. The catheterized animals were further subjected to mSNI surgeries only in the case of the presentation of normal behaviors to punctuate mechanical stimulus.

The mSNI surgeries were performed as described above. At the beginning of the fourth day after mSNIs, the animals were slightly re-anesthetized with isoflurane, and the self-made externalization devices of the intrathecal bolus delivery systems were disinfected with an aqueous iodophor solution. With the help of two pairs of small forceps, the infusion guide cannula in the externalization device was uncapped. The intrathecal bolus delivery system was first flushed with 20 μL of sterile normal saline at 37 °C, which was loaded into a sterile 50-μL Hamilton microinjection syringe with a PE-50 tubing adapter of 2-cm length. Then, 10 μL of mouse anti-αβTCR monoclonal PAbs in sterile PBS (1:5; clone R73, Bio-Rad Laboratories Inc., MCA53GA) was loaded into another sterile 50-μL Hamilton microinjection syringe with a PE-50 tubing adapter. The suppressive anti-αβTCR antibodies were infused into the subarachnoid space via the intrathecal bolus delivery system. The intrathecal bolus delivery system was further flushed twice with 20 μL of sterile normal saline at 37 °C. The infusion guide cannula was re-capped, and the externalization device was disinfected. The animal was allowed to recover before the returning to its housing cage. The intrathecal bolus application of suppressive anti-αβTCR antibodies was repeated every day until the eighth day after mSNIs. For animals of control group, the same amount of isotype control IgGs was intrathecally dosed at each day.

In order to confirm targeted suppression of CD4+ αβ T cells in lumbar dorsal root leptomeninges through chronic intrathecal delivery of the suppressive anti-αβTCR antibodies, another cohort of animals (*n* = 5 animals per group) were subjected to single intrathecal application of the suppressive anti-αβTCR antibodies or the corresponding control IgGs at the beginning of the fifth day after mSNIs. At the end of the same day, slide-mounted sections (10-μm thick) of L4 DRGs with the attaching proximal DRs were prepared and processed for single fluorescent immunolabeling against αβTCR, CD4, and CD8 as described above. In the leptomeninges covering the proximal L4 DRs at the DR portions of the SAAs, αβ T cells, CD4+ cells, and CD8+ cells were imaged and quantified as described above.

### Sensory testing for pain behaviors of mechanical allodynia

Pain behaviors of mechanical allodynia for animals were specifically characterized by the von Frey hair (VFH) test on the glabrous sural nerve skin territories of both hindpaws, i.e., the lateral plantar surfaces (Fig. 5a). For prior lymphadenectomized or sham-operated animals (*n* = 9 animals per group for LLNs and *n* = 6 animals per group for other lymph nodes), the time points are designed as the following: before lymphadenectomy, before mSNI, every day during the first week post mSNI, and at 10, 12, and 14 days post mSNI. For animals with chronic intrathecal injection of mouse anti-αβTCR monoclonal antibodies or the corresponding isotype control IgGs (*n* = 5 animals per group), the time points are designed as the following: before lumbar intrathecal catheterization, before mSNI, and at 3, 4, 5, 6, 7, 8, 9, 10, 12, and 14 days post mSNI (Fig. 6e). One week before lymphadenectomy or lumbar intrathecal catheterization, all the animals were allowed for habituation to experimental devices and manipulations for sensory tests with nociceptive baseline threshold measurement. All the behavioral tests were performed by an individual, who was blinded to the objectives of the testing, but trained in the correct execution of the behavioral tests.

As we previously described [45], the 50% paw withdrawal threshold (PWT) in response to a punctuate mechanical stimulus was measured by a series of VFHs (DanMic Global; California, USA) with the up-down method beginning with the 2.0-g hair. The von Frey filaments were perpendicularly applied to the lateral plantar surfaces of the hindpaws with enough force to cause slight buckling against the hindpaw and held for about 6–8 s. Lifting or flinching of the stimulated foot was recorded as a positive response. If rats withdrew the paws from all filaments, the 50% PWT was assigned 0.2 g; if rats did not withdraw to any of the filaments, the 50% PWT was assigned 15 g. The 50% response threshold of the hind paws for each rat was calculated by the formula: $50\%\mathrm{threshold}=\frac{10^{\chi_f+ k\delta}}{10000}$, where *X*_*f*_ = value (in log units) of the final VFH used; *k* = tabular value for the pattern of positive/negative responses; and *δ* = 0.224.

### Fluorescent immunohistochemistry of somatotopical glial and neuronal activation in spinal cords

At the designated time points (*n* = 5 animals per group at each time point), transverse frozen sections of L4 SCs (10- or 50-μm thick) from prior lymphadenectomized or sham-operated animals for the LLNs were prepared and processed for fluorescent IHC and imaging as we previously described [45, 46]. Free-floating thick sections of L4 SCs before and after mSNIs (five sections per sample) were subjected to GFAP or Iba1 single immunolabeling: mouse monoclonal anti-GFAP (1:5000, Proteintech, 60190-1-Ig; Wuhan, Hubei, China) and rabbit polyclonal anti-Iba1 (1:1000, Proteintech, 10904-1-AP) [46]. For the quantitative analysis of somatotopically determined IHC staining, the injured tibial innervation territories and the intact sural innervation territories of L4 SC-DH gray matter were determined as previously reported [52] (Fig. 1a). In brief, with the help of the stage navigator tool in Olympus cellSens Dimension software, the dorsolateral and dorsomedial edges of L4 SC-DH gray matter were first determined on the overview images. Then, along the mediolateral axis, the injured tibial innervation territories were identified as approximately the medial 45% portion and the intact sural innervation territories as the lateral one-third portion. Fluorescent Z-stack images (two fields/section) were taken using a × 20X (NA = 0.75) UPLSAPO objective across a defined area of interest (laminae I–III) in the injured tibial innervation territories and the intact sural innervation territories of ipsilateral and contralateral L4 SC-DH gray matter (Fig. 1a). Furthermore, fluorescent Z-stack images of higher magnification for representative GFAP-positive astrocytes (at least five cells per field of × 20 objectives) were taken using a × 60 (NA = 1.42) PLAPON oil-immersion objective. Fiji software was used to quantify the number of Iba1 or GFAP-positive microglia or astrocytes, percentage areas of immunoreactive signals relative to the target areas, and the mean pixel intensities (MPIs) of the positive signal of glial staining in the target areas.

Slide-mounted thin sections of L4 SCs before and 7 days after mSNIs (five sections per sample) were subjected to simultaneous NeuN/PKCγ double immunolabeling: mouse monoclonal anti-NeuN (1:500, Abcam, ab104224) and goat polyclonal anti-PKCγ (1:200, Abcam, ab71558) [45]. Images for laminae I–III of the gray matter within ipsilateral and contralateral SC-DHs were taken using a × 10 (NA = 0.40) UPLSAPO objective across the whole mediolateral axis. Fiji software was used to quantify the MPIs of the positive signal of neuronal PKCγ staining at the inner lamina II of the intact sural innervation territories in L4 SC-DH gray matter [52]. For both spinal glial and neuronal activation, representative images of almost the same anatomical localization in the matched sections were blindly selected for further processing and the assembly of the figures of this publication [46].

### Statistical analysis

All the quantitative data were presented as mean ± S.E.M. Statistical comparisons were made with two-way repeated-measures analysis of variance (2W-RM ANOVA) followed by Dunn’s post-test or independent *T* test. For all data analysis, *P* value less than 0.05 was considered as statistically significant.

## Results

### Mapping of αβ T cells along the somatosensory pathways after adult rat mSNIs

Seven days after mSNIs or the corresponding sham operations, we used αβTCR chromogenic and fluorescent immunolabeling to map αβ T cells from the skin up to the SC along the somatosensory pathways (Fig. 1a), which specifically transmit mechanical allodynia on the glabrous sural skin territories of the ipsilateral hindpaws (see Additional file 1: Figure S1B, C). In sham-operated animals, very few, if any, αβ T cells were occasionally observed in all the tissues examined in the present study (Fig. 1b, c; see Additional files 2 and 5: Figures S2 and S5).

Compared with sham-operated animals, αβTCR^+^ cells with morphological features of T cells (lobular or U-shaped large nuclei) are obviously present de novo in the pia and arachnoid mater covering either the proximal L4 DRs at the DR portions of the SAAs or the DRG portions of the SAAs 7 days after mSNIs (Fig. 1b; see Additional file 2: Figure S2A). There were no obvious αβ T cells in the parenchyma of L4 DRs and DRGs (Fig. 1b; see Additional file 2: Figure S2A). Further mapping studies across the whole courses of L4 DRs showed that 7 days after mSNIs, αβ T cells significantly entered into the pia mater but not the parenchyma of the middle and distal portions of L4 DRs (Fig. 1b; see Additional file 2: Figure S2A). We also observed a significant number of αβ T cells in the pia maters perforating in the parenchyma of the proximal L4 DRs 7 days after mSNIs (see Additional file 3: Figure S3). Therefore, 7 days after mSNIs, αβ T cells robustly infiltrate into the leptomeninges across the whole length of the lumbar DRs in the somatosensory pathways transmitting mechanical allodynia on the glabrous sural skin territories. By contrast, 7 days after mSNIs, there were no αβ T cells in the intact sural nerves and the glabrous sural skins from the ipsilateral hindlimbs or hindpaws (Fig. 1c; see Additional file 2: Figure S2B). For the cell-body-rich areas of L4 DRGs ipsilateral to the injured tibial nerves, there were also no obvious αβ T cells 7 days after mSNIs (Fig. 1c; see Additional file 2: Figure S2B). Moreover, minimal or no αβ T cells were observed in the parenchyma or the pia maters of L4 SC-DHs 7 days after mSNIs (Fig. 1c; see Additional file 2: Figure S2B).

We further quantitatively profiled the temporal dynamics of αβ T cell infiltration into L4 DR leptomeninges after mSNIs. After mSNIs, these T cells were shown to robustly enter into the leptomeninges covering the proximal L4 DRs at the DR portions of the SAAs, beginning at the third day, intensifying at the fifth day, peaking at the seventh day, and disappearing largely at the 14th day (Fig. 1d; see Additional file 4: Figure S4). Taken together, these results above indicated that during the sub-acute phase after mSNIs, antigen-specific αβ T cells selectively infiltrate into the leptomeninges of the lumbar DRs along the somatosensory pathways for the transmission of mechanical allodynia on the glabrous sural skin territories.

The proximal and distal stumps of the injured tibial nerves from the ipsilateral hindlimbs and the glabrous tibial skins from the ipsilateral hindpaws were also examined in this neuropathic pain model (Fig. 1a). Potential CD4+ αβ T cells there might lead to an inflammatory microenvironment and might directly or indirectly sensitize the nearby intact PSNs with their peripheral afferent axons in the intact sural nerves, which transmitted mechanical allodynia on the glabrous sural skin territories [53, 54]. Consistent with the infiltration of αβ T cells into a variety of injured nerves [11, 21–25], αβ T cells were shown to significantly enter into both the proximal and distal stumps of the injured tibial nerves (see Additional files 2 and 5: Figures S2C and S5A1, A2 B1, B2). For the hindpaw glabrous skins innervated by the injured tibial nerves, we did not observe any αβ T cells 7 days after mSNIs (see Additional files 2 and 5: Figures S2C and S5C1, C2).

### The molecular identity of αβ T cells infiltrating into the lumbar DR leptomeninges after mSNIs

**Fig. 2:**
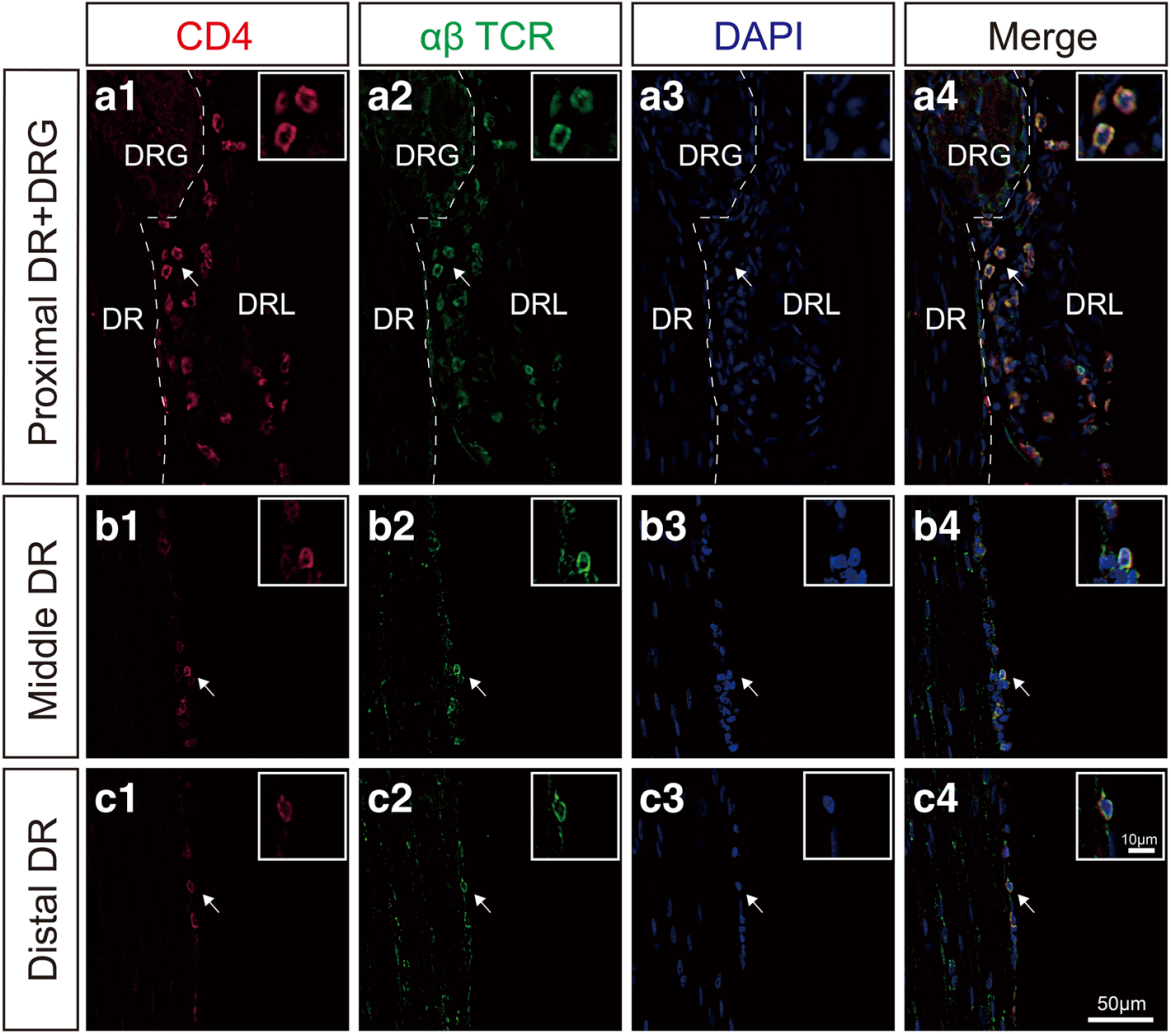
The molecular identity of αβ T cells infiltrating into the lumbar DR leptomeninges 7 days after mSNIs. CD4 and αβTCR double staining of the L4 DR leptomeninges at the DRG and the proximal DR (A1–A4), the middle DR (B1–B4), and the distal DR (C1–C4). DR dorsal root, DRG dorsal root ganglion, DRL dorsal root leptomeninge

We further characterized the molecular identity of αβ T cells infiltrating into the lumbar DR leptomeninges 7 days after mSNIs. CD4/αβTCR fluorescent double labeling results demonstrated that the vast majority of αβ T cells (98.32 ± 0.54%) are CD4 positive in the leptomeninges covering the proximal L4 DRs at the DR portions of the SAAs (Fig. 2a1–a4). Furthermore, CD8/αβTCR fluorescent double labeling results demonstrated that there are few, if any, CD8-positive cells among the αβ T cell population present in the same area (see Additional file 6: Figure S6A1–A4) and the leptomeninges covering the middle portions of L4 DRs (see Additional file 6: Figure S6b1-b4). For the other three areas of the lumbar DR leptomeninges, CD4/αβTCR fluorescent double labeling results also showed that almost all the αβ T cells are CD4 positive (Fig. 2B1–B4, C1–C4). Similarly, 5 days after mSNIs, the vast majority of αβ T cells are CD4 positive but CD8 negative in the leptomeninges covering the proximal L4 DRs at the DR portions of the SAAs (see Additional file 7: Figure S7). Therefore, these results indicated that CD4+ αβ T cells infiltrate into the lumbar DR leptomeninges during the sub-acute phase after mSNIs.

### CD4+ αβ T cell infiltration dynamics in the lumbar DR leptomeninges are specifically determined by the lumbar lymph nodes after mSNIs

**Fig. 3:**
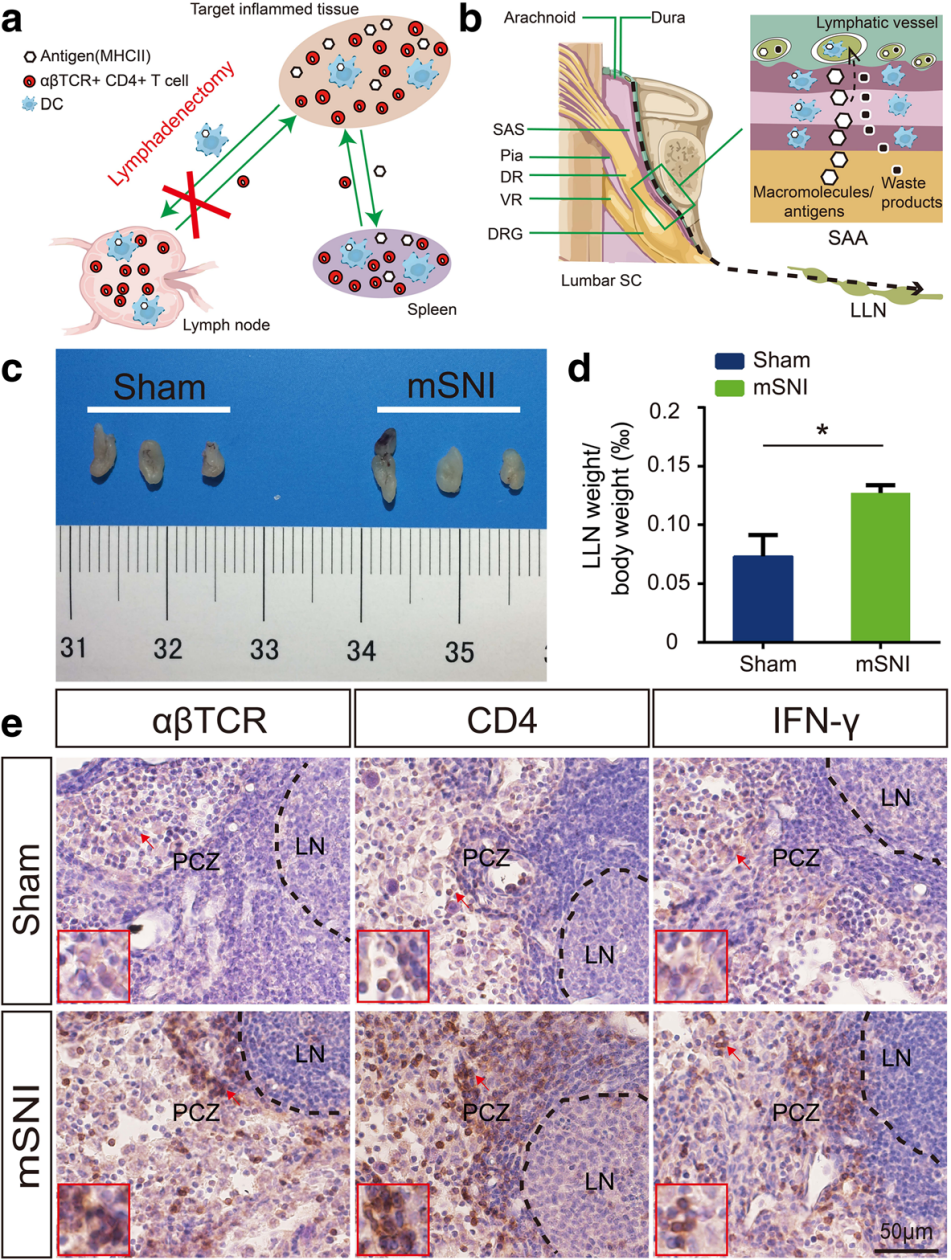
CD4+ αβ T cell responses in the lumbar lymph nodes 7 days after adult rat mSNIs. **a** Schematic presentation of the lymphadenectomy for region-specific targeting of CD4+ αβ T cells in the inflamed tissues. CD4+ αβ T cells in the spleen are broadly activated with blood-borne, MHC II-dependent pathogenic antigens from all the inflamed tissues; these T cells hence infiltrate into not only the target inflamed tissues but also other inflamed tissues. By contrast, CD4+ αβ T cells in the local lymph nodes are specifically activated with MHC II-dependent pathogenic antigens from the target inflamed tissues; these T cells hence selectively infiltrate into the target inflamed tissues. Therefore, these features rationalize the lymphadenectomy as a satisfactory method for region-specific targeting of CD4+ αβ T cells in the target inflamed tissues. **b** Schematic illustration of the LLNs as the specific local lymph nodes to drain possible pathogenic antigens in the cerebrospinal fluid (CSF) compartment at the lumbar vertebral levels. **c**, **d** Gross morphologies (**c**) and relative weights (**d**) of LLNs from mSNIed and sham-operated rats (*n* = 5/group). *P < 0.05, independent Student’s *t* test. **e** Reactive changes of CD4+ αβ T cells in LLNs from mSNIed and sham-operated rats (*n* = 5/group). DC dendritic cell, DR dorsal root, DRG dorsal root ganglion, LLN lumbar lymph node, LN lymphoid nodule, M medulla, mSNI modified spared nerve injury, PCZ paracortical zone, SAA subarachnoid angle, SAS subarachnoid space, SC spinal cord, VR ventral root

Then, we intended to specifically ascertain the roles of L4 DR leptomeningeal CD4+ αβ T cells in the development of chronic mechanical allodynia after mSNIs. This task requires region-specific targeting of antigen-specific and MHC II-restricted CD4+ αβ T cells in the target inflamed tissues, which cannot be accomplished by current genetic or pharmacological approaches at the whole-body level and splenectomy [18]. It is well known that apart from the spleen as the non-specific origin for CD4+ αβ T cells in all the inflamed tissues, CD4+ αβ T cells in the target inflamed tissues are specifically derived from the local lymph nodes, which are responsible for the specific drainage of pathogenic antigens in these stressed tissues (Fig. 3a) [55]. Moreover, the selective activation of CD4+ αβ T cells among the immune cells in the spleens after peripheral nerve injuries implied a similar pattern of immune cell activation in the local lymph nodes, i.e., selective activation of CD4+ αβ T cells. Compared with genetic or pharmacological approaches and splenectomy [18], lymphadenectomy to these lymph nodes has the ability to selectively disrupt CD4+ αβ T cell infiltration dynamics specifically in the target inflamed tissues after nerve injuries (Fig. 3a) [48, 49]. Therefore, the lymphadenectomy provides a satisfactory method for region-specific targeting of CD4+ αβ T cells in the target inflamed tissues after nerve injuries [48, 49]. Given that, we first identified the origin in local lymph nodes for CD4+ αβ T cells present in L4 DR leptomeninges after mSNIs.

Significant infiltration of CD4+ αβ T cells in L4 DR leptomeninges after mSNIs indicated the de novo release of potential pathogenic antigens into the CSF compartment related to L4 DRs after mSNIs. It has been suggested that the LLNs are responsible for the drainage of possible pathogenic antigens in the CSF compartment at the lumbar vertebral levels via the lymphatic vessels within the dura mater there (Fig. 3b) [12, 18, 37–41, 47, 48]. Hence, the activation of CD4+ αβ T cells was supposed to take place in the LLNs after mSNIs. In fact, our results showed that 7 days after mSNIs, despite the absence of obvious hypertrophy (Fig. 3c), the relative weights of LLNs to the body weights were significantly increased (Fig. 3d). More importantly, 7 days after mSNIs, CD4+ αβ T cells undergo robust activation, proliferation, and IFNγ-positive Th1 inflammatory polarization in PCZs of LLNs (Fig. 3e). The CD4+ αβ T cell responses in LLNs after mSNIs further implied the antigen-draining capacity of these lymph nodes for potential autoantigens released into the CSF compartment related to L4 DRs after mSNIs. These results therefore suggested LLNs as the specific origins in peripheral lymphoid organs for L4 DR leptomeningeal CD4+ αβ T cells.

**Fig. 4:**
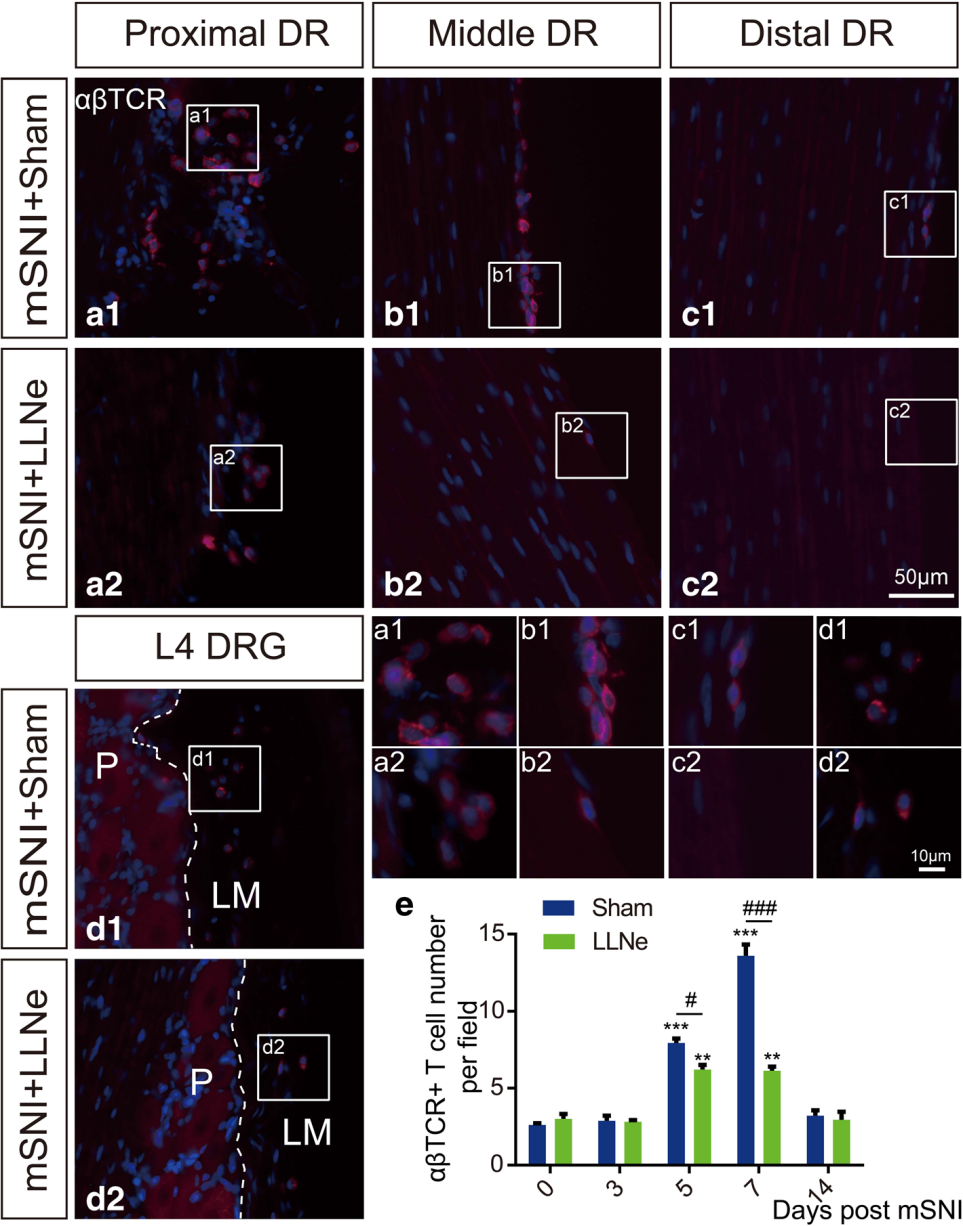
Alpha beta T cell infiltration into the lumbar DR leptomeninges are specifically determined by LLNs after adult rat mSNIs. (**A1**–**D1**, **A2**–**D2**) Alpha beta T cell infiltration into the whole length of the L4 DR leptomeninges 7 days after mSNIs in prior lymphadenectomized or sham-operated animals to LLNs (*n* = 5/group). **e** Temporal dynamics of αβ T cell entry into the leptomeninges covering the proximal L4 DRs at the DR portions of the subarachnoid angles before and after mSNIs in prior lymphadenectomized or sham-operated animals to LLNs (*n* = 5/group). ^**^P < 0.01, ^***^P < 0.001; after versus before mSNIs. ^#^*P* < 0.05, ^###^*P* < 0.001; lymphadenectomy versus sham operation. DR dorsal root, DRG dorsal root ganglion, DRL dorsal root leptomeninges, LLN lumbar lymph node, LM leptomeninges, mSNI modified spared nerve injury, P parenchyma

Then, we performed prior lymphadenectomy to LLNs 7 days before mSNIs and then assessed αβ T cell infiltration into L4 DR leptomeninges after mSNIs. In comparison with sham-operated animals, the removal of LLNs significantly reduced the number of αβ T cells in the leptomeninges across the whole length of the lumbar DRs 7 days after mSNIs (Fig. 4A–D). This indicated that a large number of L4 DR leptomeningeal αβ T cells are specifically derived from LLNs. Further quantitative profiling results showed that in comparison with sham-operated animals, the removal of LLNs significantly reduced the number of αβ T cells in L4 DR leptomeninges during the sub-acute phase after mSNIs (Fig. 4E; see Additional file 9: Figure S9). There were indeed some remaining αβ T cells in the L4 DR leptomeninges of these lymphadenectomized animals (Fig. 4; see Additional file 9: Figure S9); the spleens might be the origins for these T cells. In addition, in comparison with sham-operated animals, the removal of LLNs had no obvious effects on the number of αβ T cells in the proximal or distal segments of the injured tibial nerves 7 days after mSNIs (see Additional file 10: Figure S10). Therefore, αβTCR^+^CD4+ T cell infiltration dynamics in the lumbar DR leptomeninges are specifically determined by the LLNs after mSNIs.

### Prior lymphadenectomy to lumbar lymph nodes reduces the development of mSNI-induced chronic mechanical allodynia

**Fig. 5:**
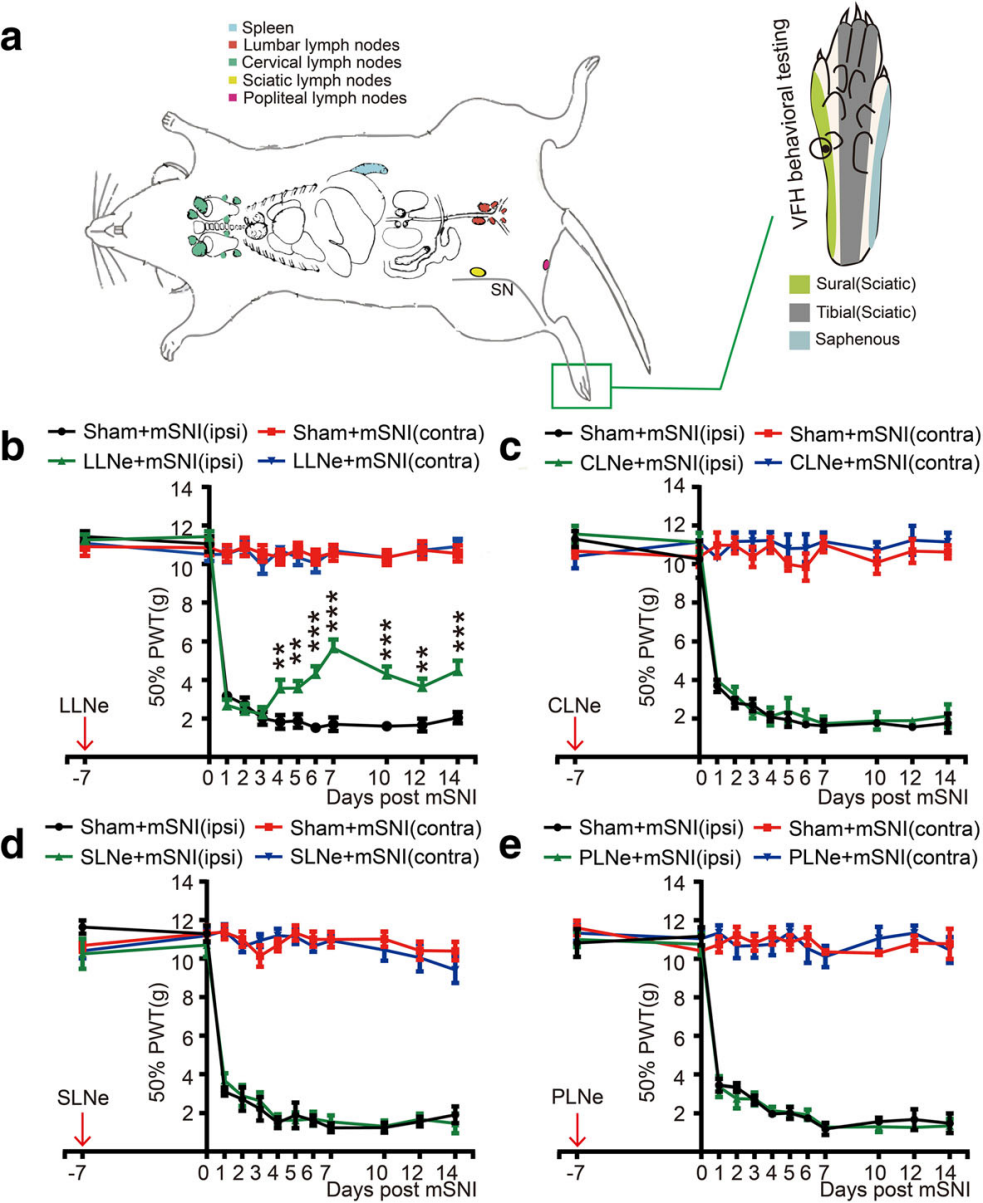
The effects of prior lymphadenectomies on the development of mechanical allodynia after adult rat mSNIs. **a** Schematic illustration of prior lymphadenectomies to the local lymph nodes along the somatosensory pathways 7 days before mSNIs on the right hindlimbs, and VFH behavioral testing for mechanical allodynia on the glabrous sural skin areas, i.e., the lateral plantar surfaces of the hindpaws. **b**–**e** Temporal dynamics of 50% PWTs (g) for both ipsilateral (ipsi) and contralateral (contra) hindpaws before and after mSNIs in lymphadenectomized or sham-operated animals to LLNs (**b**; *n* = 9/group), CLNs (**c**; *n* = 6/group), SLNs (**d**; *n* = 6/group), and PLNs (**e**; *n* = 6/group). ^**^P < 0.01; ^***^P < 0.001; lymphadenectomy versus sham operation for ipsilateral hindpaws. CLN cervical lymph node, LLN lumbar lymph node, mSNI modified spared nerve injury, PLN popliteal lymph node, PWT paw withdrawal threshold, SLN sciatic lymph node, SN sciatic nerve, VFH von Frey hair

With the specific coupling between LLNs and L4 DR leptomeninges for CD4+ αβ T cell dynamics following mSNIs in mind, we assessed the roles of these T cells in the development of chronic mechanical allodynia after mSNIs. We performed prior lymphadenectomy against LLNs to specifically reduce the number of L4 DR leptomeningeal CD4+ αβ T cells following mSNIs and then assessed temporal dynamics of mSNI-induced mechanical allodynia in these lymphadenectomized or sham-operated animals (Fig. 5a). We also included prior lymphadenectomy to CLNs (Fig. 5a), since these nodes have been reported to possibly drain some portions of potential antigens in the CSF compartment at the lumbar vertebral levels [48] and might be another specific origin in peripheral lymphoid organs for CD4+ αβ T cells in the lumbar DR leptomeninges. Moreover, we performed prior lymphadenectomy to sciatic or popliteal lymph nodes (Fig. 5a), which are responsible for the drainage of possible autoantigens in the injured tibial nerves [21, 22, 28, 47] and therefore should be the specific origins in peripheral lymphoid organs for αβ T cells in the injured tibial nerves.

In lymphadenectomized animals to LLNs, the development of mSNI-induced mechanical allodynia remained until to the third day after mSNIs (the acute phase) but significantly attenuated from the fourth day to the follow-up after mSNIs (Fig. 5b). The observed long-lasting disruptions in the development of mechanical allodynia from the sub-acute to chronic phase indicated that prior lymphadenectomy to LLNs reduces the transition from acute to chronic mechanical allodynia after mSNIs. By contrast, in lymphadenectomized animals to CLNs, the development of mSNI-induced mechanical allodynia did not show any significant changes in comparison with sham-operated animals (Fig. 5c). This implied that CLNs might not drain any possible autoantigens in the CSF compartment at the lumbar vertebral levels after mSNIs and that CD4+ αβ T cells in L4 dorsal root leptomeninges following mSNIs could not be derived from these lymph nodes.

In addition, in comparison with the corresponding sham-operated animals, our results showed that the removal of sciatic or popliteal lymph nodes did not alter the development of mSNI-induced mechanical allodynia (Fig. 5d, e). Our results further showed that prior lymphadenectomies to SLNs or PLNs could significantly reduce the number of αβ T cells in proximal and distal stumps of the injured tibial nerves (see Additional file 11: Figure S11). Therefore, these results indicated that αβ T cells robustly present in proximal and distal stumps of the injured tibial nerves are not necessary for the development of chronic mechanical allodynia after mSNIs.

Taken together, these results mentioned above demonstrated that prior lymphadenectomy to LLNs specifically attenuates the development of mSNI-induced chronic mechanical allodynia and therefore implied that CD4+ αβ T cells, selectively infiltrating into the L4 DR leptomeninges after mSNIs, contribute to the transition from acute to chronic mechanical allodynia after nerve injuries.

### Intrathecal application of the suppressive anti-αβTCR antibodies reduces the development of mSNI-induced chronic mechanical allodynia

**Fig. 6:**
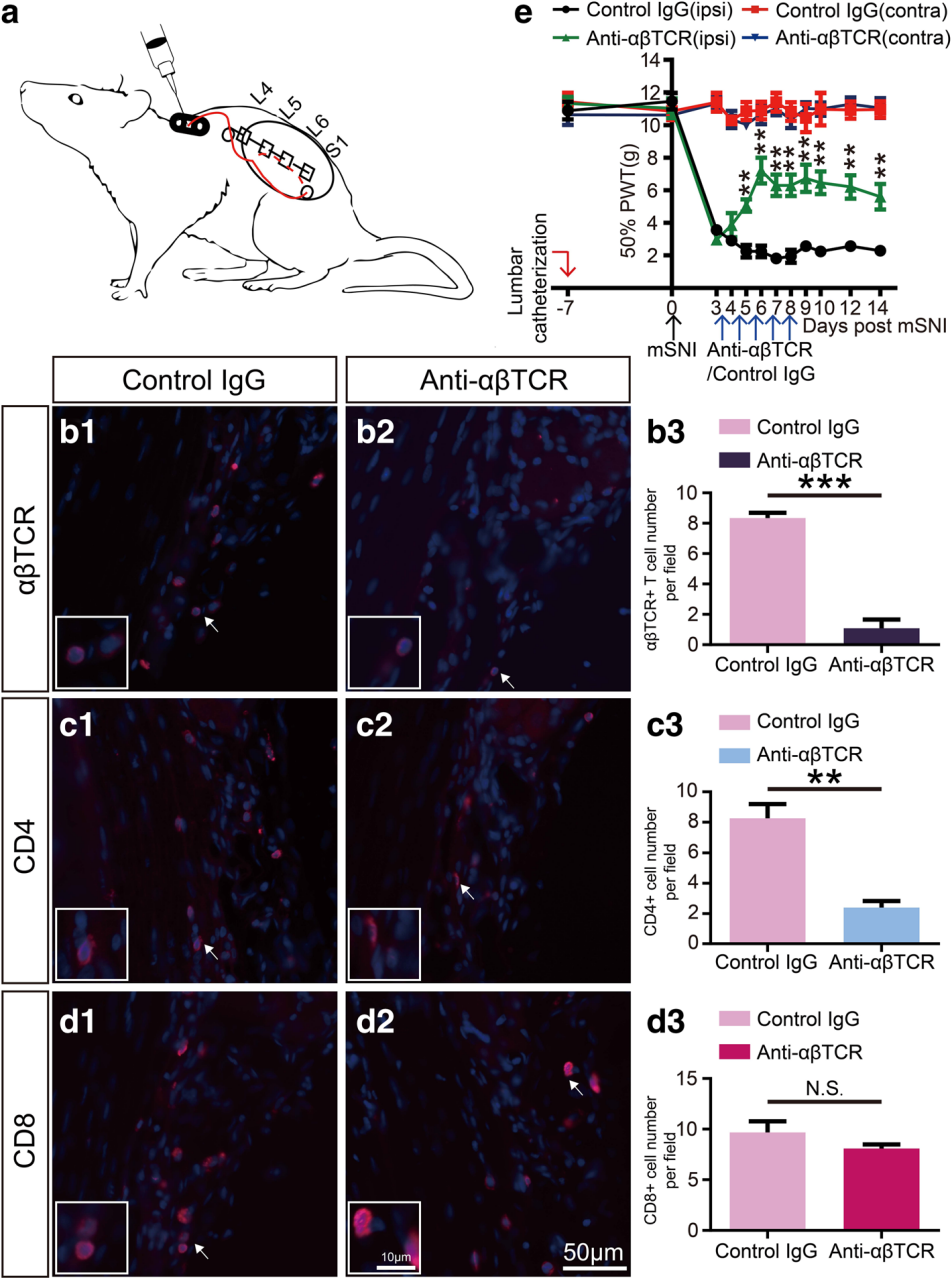
The effects of chronic intrathecal application of the suppressive anti-αβTCR antibodies on the development of mechanical allodynia after adult rat mSNIs. **a** Schematic illustration of lumbar catheterization and externalization with the self-made intrathecal bolus delivery system for repeated intrathecal injection. **b1**-**b3**, **c1**-**c3**, **d1**-**d3** Representative images and quantitative presentations for the numbers of αβ T cells (**b1**-**b3**), CD4+ cells (**c1**–**c3**), and CD8+ cells (**d1**–**d3**) in the leptomeninges covering the proximal L4 DRs at the DR portions of the SAAs 24 h after acute intrathecal injection of mouse anti-αβTCR monoclonal antibodies or the corresponding isotype control IgGs at the beginning of the fifth day after mSNIs (*n* = 5/group). **e** Temporal dynamics of 50% PWTs (g) for both ipsilateral (ipsi) and contralateral (contra) hindpaws before and after mSNIs in animals with chronic intrathecal injection of mouse anti-αβTCR monoclonal antibodies or the corresponding isotype control IgGs (*n* = 5/group). ^*^P < 0.05, ^**^P < 0.01, ^***^P < 0.001; anti-αβTCR versus control IgG. mSNI modified spared nerve injury, PWT paw withdrawal threshold

For more direct suppression of CD4+ αβ T cells in lumbar dorsal root leptomeninges after mSNIs, we employed repeated intrathecal application of the suppressive anti-αβTCR antibodies during the sub-acute phase after mSNIs (Fig. 6A, E). The mouse anti-αβTCR monoclonal PAbs (clone R73) has been extensively used for the suppression of αβ T cells in vivo, either through targeted depletion of αβ T cells or the suppression of proinflammatory cytokine release from activated T cells [56, 57]. In fact, at the beginning of the fifth day after mSNIs, single intrathecal application of the suppressive anti-αβTCR antibodies almost completely depleted αβ T cells at the end of the same day (Fig. 6B1–B3). Meanwhile, CD4+ cells, but not CD8+ cells, were almost completely depleted by single intrathecal delivery of this anti-αβTCR antibody (Fig. 6C1–C3, D1–D3). These results further indicated that CD4+ αβ T cells, but not CD8+ αβ T cells, selectively infiltrate into lumbar dorsal root leptomeninges in the subarachnoid space during the sub-acute phase after mSNIs. Hence, during the sub-acute phase after mSNIs, long-term intrathecal application of the suppressive anti-αβTCR antibodies could specifically deplete CD4+ αβ T cells in lumbar dorsal root leptomeninges.

During the sub-acute phase after mSNIs, chronic intrathecal injection of the suppressive anti-αβTCR antibodies, but not mouse isotype control IgGs, significantly attenuated the development of chronic mechanical allodynia after mSNIs (Fig. 6E). This suppressive effect was obviously revealed in three of five animals at the fourth day after mSNIs, became more significant and stable at the fifth day, and peaked at the sixth day. Moreover, this inhibitory effect was long-lasted during the follow-up until the 14th day after mSNIs, in spite of the cessation of intrathecal injection of the suppressive anti-αβTCR antibodies at the eighth day. These results further indicated that CD4+ αβ T cells, selectively infiltrating into the L4 DR leptomeninges after mSNIs, contribute to the transition of acute mechanical allodynia to a chronic state after nerve injuries.

### Prior lymphadenectomy to lumbar lymph nodes attenuates mSNI-induced spinal activation of glial cells and PKCγ^+^ excitatory interneurons

It has been well established that robust spinal activation of glial cells, microglia, and astrocytes, in particular, are essential for the development of chronic mechanical allodynia after peripheral nerve injuries, through central sensitization of neural circuits in SC-DHs specific for chronic mechanical allodynia, such as aberrant activation of PKCγ^+^ excitatory interneurons in the inner lamina II [3–5, 8–10]. Therefore, we further assessed the potential inhibitory effects of CD4+ αβ T cells infiltrating into the L4 DR leptomeninges on mSNI-induced glial and neuronal changes in L4 SC-DHs. We used prior lymphadenectomy of LLNs as the method to specifically target CD4+ αβ T cells infiltrating into the L4 DR leptomeninges after mSNIs.

**Fig. 7:**
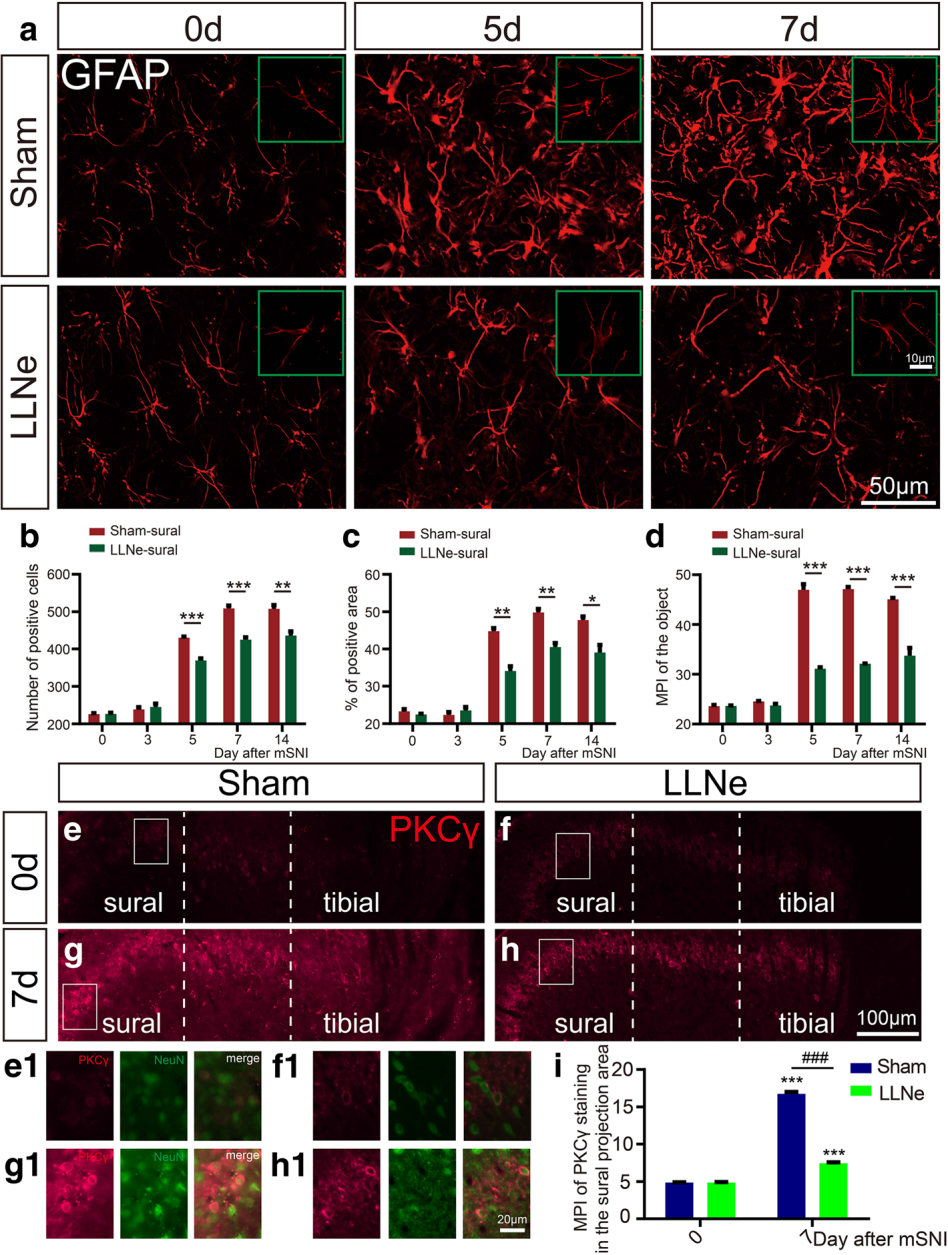
The effects of prior lymphadenectomy to lumbar lymph nodes on mSNI-induced spinal sensitizations. **a**–**d** Representative images and quantitative presentations for temporal dynamics of astrocyte activation in the sural projection areas of L4 SC-DHs before and after mSNIs in lymphadenectomized or sham-operated animals to LLNs (*n* = 5/group). ^*^P < 0.05; ^**^P < 0.01; ^***^P < 0.001; lymphadenectomy versus sham operation. **e**–**h** Somatotopically PKCγ expression in PKCγ^+^ excitatory interneurons in the inner lamina II within the gray matter of L4 spinal cord dorsal horns before mSNIs and 7 days after mSNIs in lymphadenectomized or sham-operated animals to LLNs (*n* = 5/group). **i** MPI of PKCγ staining in the sural projection areas of L4 SC-DHs before mSNIs and 7 days after mSNIs in lymphadenectomized or sham-operated animals to LLNs (*n* = 5/group). ^***^P < 0.001; after versus before mSNIs. ^###^*P* < 0.001; lymphadenectomy versus sham operation. LLN lumbar lymph node, mSNI modified spared nerve injury, MPI mean pixel intensity

In comparison with sham-operated animals, mSNI-induced spinal activation of glial cells, especially astrocytes, was shown to be significantly attenuated in these lymphadenectomized animals during the sub-acute phase, not only in the injured tibial innervation territories but also in the intact sural innervation territories (Fig. 7a–d; see Additional files 12, 13, 14, 15: Figures S12–S15). Moreover, for these lymphadenectomized animals 7 days after mSNIs, mSNI-induced robust upregulation of PKCγ neuronal expression at the inner lamina II of L4 SC-DHs was also demonstrated to be significantly reduced in both the injured tibial innervation territories and the intact sural innervation territories (Fig. 7e–i). Therefore, these results showed that prior lymphadenectomy of LLNs reduces mSNI-induced spinal activation of glial cells and PKCγ^+^ excitatory interneurons. This also implied that antigen-specific and MHC II-restricted CD4+ αβ T cells in the leptomeninges of the L4 DRs play a role in glial activation and neuronal sensitization in SC-DHs after mSNIs.

## Discussion

Antigen-specific and MHCII-restricted CD4+ αβ T cells have been shown or suggested to play an important role in the transition from acute to chronic mechanical allodynia after peripheral nerve injuries. However, it is still largely unknown where these T cells infiltrate along the somatosensory pathways transmitting mechanical allodynia to initiate the development of chronic mechanical allodynia after nerve injuries. In the present study, we provide the first noteworthy evidence that CD4+ αβ T cells selectively infiltrate into the DR leptomeninges of the somatosensory pathways transmitting mechanical allodynia and contribute to the transition of acute mechanical allodynia to a chronic state after peripheral nerve injuries.

### The DR leptomeninges as the neuroimmune interface for CD4+ αβ T cells to initiate the development of chronic mechanical allodynia after peripheral nerve injuries

Previous studies from McLachlan et al. [26–28] provided some suggestive evidence that after adult rat SSNL or CCI to the sciatic nerves, αβ T cells robustly infiltrate into the leptomeninges of the SAAs at the transitional zone between the lumbar DRs and DRGs. In our present study, we characterized this potential phenomenon in the mSNI pain model. For the first time, we found that αβ T cells robustly and selectively infiltrate into the leptomeninges across the whole courses of the lumbar DRs in the somatosensory pathways transmitting mechanical allodynia. Moreover, we observed that almost all the αβ T cells there are CD4 positive. To establish the universality of this phenomenon, it should be further characterized after different conditions of peripheral nerve injuries. Even though, we could conclude that antigen-specific and MHC II-restricted CD4+ αβ T cells selectively enter into the DR leptomeninges along the somatosensory pathways for the transmission of mechanical allodynia after peripheral nerve injuries.

The DR meninges are anatomically the lateral extension of spinal meninges and presumably have the same embryonic origin as that of spinal meninges [37–42]. This notion was further supported by histological evidences that the DR meninges potentially have similar immunological elements to those in cerebrospinal meninges [39–42]. There are MHCII-expressing macrophages and dendritic cells, which line on the DR leptomeninges adjacent to the CSF compartments [39]. In the intervertebral foramens, lymphatic vessels are present in the DR dura mater and epidural tissues [40]. There is also a rich amount of blood microvessels in the DR leptomeninges [39–42]. For cerebrospinal meninges, this kind of neuroimmune microenvironment has been demonstrated to enable cerebrospinal meninges as the multifaceted neuroimmune interface for T cell interactions with the healthy or stressed CNS [36]. Given the anatomical, embryonic, and histological similarities, the DR meninges are likely to be functionally similar, if not identical, to cerebrospinal meninges for T cell interactions with the nervous system. Hence, antigen-specific and MHC II-restricted CD4+ αβ T cells selectively present in the DR leptomeninges might have functional roles in the development of chronic mechanical allodynia after peripheral nerve injuries.

We first used prior lymphadenectomy to LLNs for region-specific disruption of CD4+ αβ T cell infiltration into L4 DR leptomeninges after mSNIs. However, prior lymphadenectomy to LLNs might affect other immune cells in the LLNs, such as CD8+ αβ T cells, B cells, NK cells, and macrophages, although CD4+ αβ T cells are supposed to be selectively activated by dendritic cells in LLNs similar with those in the spleens after peripheral nerve injuries. Hence, our lymphadenectomy results can just suggest that CD4+ αβ T cells in the DR leptomeninges should have a role in the development of chronic mechanical allodynia after peripheral nerve injuries. We further employed repeated intrathecal injection of the suppressive anti-αβTCR antibodies during the sub-acute phase after mSNIs, which allowed more direct and specific suppression of CD4+ αβ T cells in lumbar dorsal root leptomeninges after mSNIs [56, 57]. Our intrathecal injection results provided the first evidence that CD4+ αβ T cells, selectively infiltrating into the L4 DR leptomeninges after mSNIs, contribute to the development of chronic mechanical allodynia after nerve injuries. Moreover, these T cells could have a role in significant activation of glial cells and PKCγ^+^ excitatory interneurons in SC-DHs, which underlie the development of chronic mechanical allodynia. Therefore, our results for the first time identified the DR leptomeninges as the new and definite neuroimmune interface for CD4+ αβ T cells to initiate the transition from acute to chronic mechanical allodynia after nerve injuries.

### The infiltration of αβ T cells into other areas along the somatosensory pathways specific for mechanical allodynia after peripheral nerve injuries

Antigen-specific αβ T cells have been conclusively found to infiltrate into the injured nerves after CCI or chronic mild compression to the sciatic nerves of adult rat or mice [11, 21, 22, 24, 25]. While the presence of αβ T cells remains to be examined in the sciatic nerves after SSNL and partial sciatic nerve ligation (PSNL), it seems that αβ T cell infiltration into the injured peripheral nerves should be a general and intrinsic process [23]. In these three pain models, these αβ T cells might have a role in the development of chronic mechanical allodynia by the sensitization of PSNs transmitting mechanical allodynia, whose peripheral afferent axons are intact and present in partially injured sciatic nerves [58]. However, this suggestion has not been directly addressed by region-specific targeting of these T cells [15]. The number of αβ T cells was shown to have no correlation with the levels of mechanical allodynia after adult rat CCI to the sciatic nerve [25]. This might suggest a dispensable role of αβ T cells in partially injured nerves for chronic transition of mechanical allodynia. This dispensability was further confirmed by our current study using the mSNI pain model. In this model, peripheral afferent axons of PSNs transmitting mechanical allodynia (the sural nerve origin) and axotomized PSNs (the tibial nerve origin) are anatomically separated along the course of the injured tibial nerves. In the present study, our results here showed robust infiltration of αβ T cells into the injured tibial nerves, rather than the intact sural nerves and the glabrous skin tissues innervated by either the sural or the tibial nerves. This excluded the possibility on the spatial scale for αβ T cells to sensitize intact PSNs of the sural nerve origin, which transmit mechanical allodynia [52, 53]. We further used prior lymphadenectomy to popliteal or sciatic lymph nodes for region-specific targeting of αβ T cells in injured tibial nerves, and our results implied the dispensability of these T cells for the development of chronic mechanical allodynia after mSNIs. Hence, αβ T cells in the injured nerves may be not necessary for the development of chronic mechanical allodynia after peripheral nerve injuries.

Along the somatosensory pathways specific for mechanical allodynia after peripheral nerve injuries, the cell-body-rich areas of DRGs have been suggested as a potential neuroimmune interface for T cells, αβ T cells in particular, to initiate the transition from acute to chronic mechanical allodynia [17, 21]. Previous studies by other groups indicated that T cells (CD3 positive) are significantly present in these regions after SNI or PSNL in adult male or female C57BL/6 mice [17, 32, 59]. However, in our present study, 7 days after mSNIs in adult male SD rats, very few, if any, αβ T cells were observed there. This implied that T cells infiltrating into the cell-body-rich areas of DRGs would be largely αβTCR-negative T cells. In contrast, a small number of αβ T cells were found to significantly infiltrate into the cell-body-rich areas of DRGs after CCI or chronic mild compression to the sciatic nerves as well as L5 SSNL in adult rats [21, 22, 24–27]. The obvious variances of antigen-specific αβ T cell infiltration there might be related to the differences of animal genetic backgrounds or immune conditioning during the life histories before nerve injuries [10, 16, 25–27] and the intrinsic distinctions of peripheral nerve injuries [24, 26–28]. However, the presence or absence of αβ T cells [24] and even the number of αβ T cells in case of significant αβ T cell infiltration [21, 22] do not correlate with the development of chronic mechanical allodynia. Hence, we can conclude, at least, that the cell-body-rich areas of DRGs are not the necessary neuroimmune interface for antigen-specific αβ T cells to initiate the development of chronic mechanical allodynia after nerve injuries.

The gray matter of SC-DHs have also been viewed as an important neuroimmune interface for T cells, including αβ T cells, to initiate the transition of acute mechanical allodynia to a chronic state after nerve injuries [12, 13, 16, 18, 27, 29–31]. However, growing bodies of evidence doubt the presence of αβ T cells, even T cells there. Firstly, a recent study [33] reported little, if any, CD2-positive cells (presumably T cells) in the SC-DHs after SNIs in adult rats, with the same experimental settings as those in a previously seminal study [13] concerning the roles of T cells of the SC-DHs in nerve injury-induced chronic mechanical allodynia. In our present study, we also did not find any convincing evidences for the presence of αβ T cells in the SC-DHs after adult rat mSNIs. Secondly, after L5 SSNL in adult male BALB/C or DBA/2 mice [12, 18], a small number of CD3-positive T cells (susceptible results per se) were found to significantly infiltrate into the SC-DHs. However, there were no T cells detected in the SC-DHs of adult male C57BL/6 mice with the same nerve injury [18]. Thirdly, while a very small number of T cells were found to significantly enter into the SC-DHs after PSNL in adult male SD or Wistar rats [29, 31], there were minimal or no T cell (CD3 positive) infiltration into the SC-DHs after PSNL in adult male C57BL/6 mice [32, 34, 35]. Finally, after CCI to the sciatic nerves of adult male rats [21, 22, 27], very low densities of αβ T cells relative to the volume of the SC-DHs were detected in the ipsilateral SC-DHs in statistical sense. Based on these results described above, the great discrepancies of T cell infiltration into the SC-DHs after nerve injuries might be also related to the differences of animal genetic backgrounds or immune conditioning during the life histories [10, 16, 25–27] before nerve injuries and the intrinsic distinctions of peripheral nerve injuries [24, 26–28]. However, chronic mechanical allodynia was still significantly developed in all the conditions of nerve injuries mentioned above. Therefore, it becomes clear, at least, that the SC-DH is not an indispensable neuroimmune interface for T cells, including αβ T cells, to initiate the development of chronic mechanical allodynia after nerve injuries.

## Conclusions

**Fig. 8:**
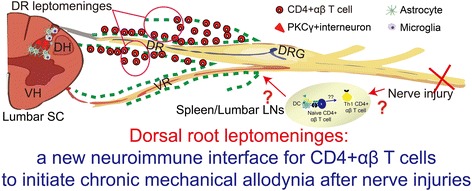
Schematic model summary for CD4+ αβ T cells to initiate the development of chronic mechanical allodynia after peripheral nerve injuries. After traumatic nerve injuries (partial injuries to the sciatic nerves here), potential unknown autoantigens were present de novo in the cerebrospinal fluid (CSF) compartment at the lumbar vertebral levels and made CD4+ αβ T cells to undergo activation, proliferation, and Th1 inflammatory polarization specifically in the corresponding antigen-draining local lymph nodes (lumbar lymph nodes here) and also in the spleen. For current unknown mechanisms, these antigen-specific and MHC II-restricted CD4+ αβ T cells selectively infiltrate into the DR leptomeninges along the somatosensory pathways transmitting mechanical allodynia. These T cells have an important role in the transition of acute mechanical allodynia to a chronic state and the corresponding glial activation and neuronal sensitization within the gray matter of spinal cord dorsal horns. The direct downstream pathogenic mechanisms for these CD4+ αβ T cells remain unknown. DC dendritic cell, DH dorsal horn, DR dorsal root, DRG dorsal root ganglion, LN lymph node, SC spinal cord, VH ventral horn, VR ventral root

The noteworthy results here provide the first evidence that inflammatory CD4+ αβ T cells (IFNγ-expressing Th1 cells) selectively enter into the DR leptomeninges along the somatosensory pathways for the transmission of mechanical allodynia; these antigen-specific and MHC II-restricted T cells contribute to the development of chronic mechanical allodynia after peripheral nerve injuries (Fig. 8). To our best knowledge, we for the first time identified the DR leptomeninges as the new and definite neuroimmune interface for CD4+ αβ T cells to initiate the transition from acute to chronic mechanical allodynia after nerve injuries. Our results here provided the anatomical basis for further insights of the roles and mechanisms of CD4+ αβ T cells, even the whole T cell population, in the development of chronic mechanical allodynia and other forms of chronic pain after nerve injuries. Our current data also extend the notion of the cerebrospinal meninges as the neuroimmune interface to the state that the DR meninges could be a functionally critical neuroimmune interface as well.

## Additional files

Additional file 1: **Figure S1.** The development of chronic mechanical allodynia after adult rat mSNIs. (A) Schematic illustration of surgical procedures for mSNIs, i.e., tibial nerve injuries, on the right hindlimbs. (B) VFH behavioral testing for mechanical allodynia on the glabrous sural skin areas, i.e., the lateral plantar surfaces of the hindpaws. (C) Temporal dynamics of 50% PWTs (g) for both ipsilateral (ipsi) and contralateral (contra) hindpaws before and after mSNIs or sham surgeries (*n* = 6/group). ^***^P < 0.001; mSNIs versus sham surgeries for ipsilateral hindpaws. mSNI: modified spared nerve injury; PWT: paw withdrawal threshold; VFH, von Frey hair. (JPEG 2492 kb)

Additional file 2: **Figure S2.** Mapping of αβ T cells along the somatosensory pathways 7 days after adult rat mSNIs or sham surgeries with chromogenic IHC for αβTCR. (A) The infiltration of αβ T cells across the whole course of the L4 DR leptomeninges 7 days after mSNIs and sham operations. (B) The infiltration of αβ T cells in the cell-body-rich areas of L4 DRGs, L4 SC-DHs, the sural nerves, and the hindpaw glabrous sural skins 7 days after mSNIs and sham operations. (C) The infiltration of αβ T cells in the proximal and distal stumps of the injured tibial nerves and the hindpaw glabrous tibial skins 7 days after mSNIs and sham operations. DR: dorsal root; DRG: dorsal root ganglion; LM: leptomeninge; mSNI: modified spared nerve injury; P: parenchyma; SC-DH: spinal cord dorsal horn; TN: tibial nerve. (JPEG 4787 kb)

Additional file 3: **Figure S3.** AβTCR^+^ T cells in the pia maters perforating in the parenchyma of the proximal L4 DRs 7 days after mSNIs (B) and sham surgeries (A). *n* = 5/group. mSNI: modified spared nerve injury. (JPEG 246 kb)

Additional file 4: **Figure S4.** The temporal dynamics of αβTCR^+^ T cell infiltration into the leptomeninges covering the proximal L4 DRs at the DR portions of the subarachnoid angles after mSNIs and sham operations (*n* = 5/group for each time point). Images in the white boxes show high magnified views of positive cells in the respective images of low magnification. (F1-F2) The corresponding staining control for D1 and D2 images by substituting primary antibodies with the corresponding isotype control IgGs. mSNI: modified spared nerve injury. (JPEG 1087 kb)

Additional file 5: **Figure S5.** The infiltration of αβ T cells in the proximal and distal stumps of the injured tibial nerves and the hindpaw glabrous tibial skins 7 days after mSNIs and sham operations. *n* = 5/group. (D–F) The corresponding staining controls for A2 image. mSNI: modified spared nerve injury; TN: tibial nerve. (JPEG 952 kb)

Additional file 6: **Figure S6.** The molecular identity of αβ T cells infiltrating into the lumbar DR leptomeninges 7 days after mSNIs. CD8 and αβTCR double staining of the L4 DR leptomeninges at the proximal DR (A1–A4) and the middle DR (B1–B4). DR: dorsal root. (JPEG 1050 kb)

Additional file7: **Figure S7.** The molecular identity of αβ T cells infiltrating into the lumbar DR leptomeninges 5 days after mSNIs. CD4 (A1–A4) or CD8 (B1–B4) and αβTCR double staining of the L4 DR leptomeninges at the proximal DR. DR: dorsal root. (JPEG 988 kb)

Additional file 8: **Figure S8.** Schematic illustration of surgical procedures for prior lymphadenectomies to LLNs (A), CLNs (B), SLNs (C), and PLNs (D) 7 days before mSNIs on the right hindlimbs. Left panel: skin incisions; right panel: the corresponding local lymph nodes (dashed circles). CLN, cervical lymph node; LLN, lumbar lymph node; PLN, popliteal lymph node; SLN, sciatic lymph node. (JPEG 2191 kb)

Additional file 9: **Figure S9.** Temporal dynamics of αβTCR^+^ T cell entry into the leptomeninges covering the proximal L4 DRs at the DR portions of the subarachnoid angles before and after mSNIs in prior lymphadenectomized or sham-operated animals to LLNs (*n* = 5/group). LLN: lumbar lymph node; mSNI: modified spared nerve injury. (JPEG 990 kb)

Additional file 10: **Figure S10.** Representative images and quantitative presentations for the numbers of αβ T cells in the proximal (A1–C1) or distal (A2–C2) stumps of the injured tibial nerves 7 days after mSNIs in prior lymphadenectomized or sham-operated animals to LLNs (*n* = 5/group). N.S., no significance; lymphadenectomy versus sham-operation. LLN: lumbar lymph node; mSNI: modified spared nerve injury. (JPEG 628 kb)

Additional file 11: **Figure S11.** Representative images and quantitative presentations for the numbers of αβ T cells in the proximal (A1–C1, D1–F1) or distal stumps (A2–C2, D2–F2) of the injured tibial nerves 7 days after mSNIs in prior lymphadenectomized or sham-operated animals to SLNs (A1–C1, A2–C2) or PLNs (D1–F1, D2–F2) (*n* = 5/group). ^**^P < 0.01; ^***^ *P* < 0.001; lymphadenectomy versus sham operation. SLN: sciatic lymph node; PLN: popliteal lymph node; mSNI: modified spared nerve injury. (JPEG 1098 kb)

Additional file 12: **Figure S12.** Representative images for temporal dynamics of astrocyte activation in the sural projection areas of L4 SC-DHs before and after mSNIs in lymphadenectomized or sham-operated animals to LLNs (*n* = 5/group). LLN: lumbar lymph node; mSNI: modified spared nerve injury. (JPEG 2303 kb)

Additional file 13: **Figure S13.** Representative images (A) and quantitative presentations (C-D) for temporal dynamics of astrocyte activation in the tibial projection areas of L4 SC-DHs before and after mSNIs in lymphadenectomized or sham-operated animals to LLNs (*n* = 5/group). ^*^P < 0.05; ^**^P < 0.01; ^***^ *P* < 0.001; lymphadenectomy versus sham operation. LLN: lumbar lymph node; mSNI: modified spared nerve injury. (JPEG 2624 kb)

Additional file 14: **Figure S14.** Representative images (A) and quantitative presentations (C-D) for temporal dynamics of microglia activation in the sural projection areas of L4 SC-DHs before and after mSNIs in lymphadenectomized or sham-operated animals to LLNs (*n* = 5/group). ^*^P < 0.05; ^**^P < 0.01; lymphadenectomy versus sham operation. LLN: lumbar lymph node; mSNI: modified spared nerve injury. (JPEG 3931 kb)

Additional file 15: **Figure S15.** Representative images (A) and quantitative presentations (C-D) for temporal dynamics of microglia activation in the tibial projection areas of L4 SC-DHs before and after mSNIs in lymphadenectomized or sham-operated animals to LLNs (*n* = 5/group). ^*^P < 0.05; ^**^P < 0.01; lymphadenectomy versus sham operation. LLN: lumbar lymph node; mSNI: modified spared nerve injury. (JPEG 3757 kb)

## Abbreviations

CatS: Cathepsin S
CCI: Chronic constriction injury
CLN: Cervical lymph node
CNS: Central nervous system
CSF: Cerebrospinal fluid
DC: Dendritic cell
DR: Dorsal root
DRG: Dorsal root ganglion
IHC: Immunohistochemistry
LLN: Lumbar lymph node
mSNI: Modified spared nerve injury
PAb: Primary antibody
PCZ: Paracortical zone
PLN: Popliteal lymph node
PNL: Partial nerve ligation
PSN: Primary sensory neuron
PSNL: Partial sciatic nerve ligation
PWT: Paw withdrawal threshold
RT: Room temperature
SAA: Subarachnoid angle
SAb: Secondary antibody
SC: Spinal cord
SC-DH: Spinal cord dorsal horn
SD: Sprague-Dawley
SLN: Sciatic lymph node
SNI: Spared nerve injury
SSNL: Selective spinal nerve ligation
VFH: von Frey hair
