# Supplementary figures and images for: CD4+ αβ T cell infiltration into the leptomeninges of lumbar dorsal roots contributes to the transition from acute to chronic mechanical allodynia after adult rat tibial nerve injuries
Source: J Neuroinflammation. 2018 Mar 15;15:81. doi: 10.1186/s12974-018-1115-7 (PMC5855984; doi:10.1186/s12974-018-1115-7)

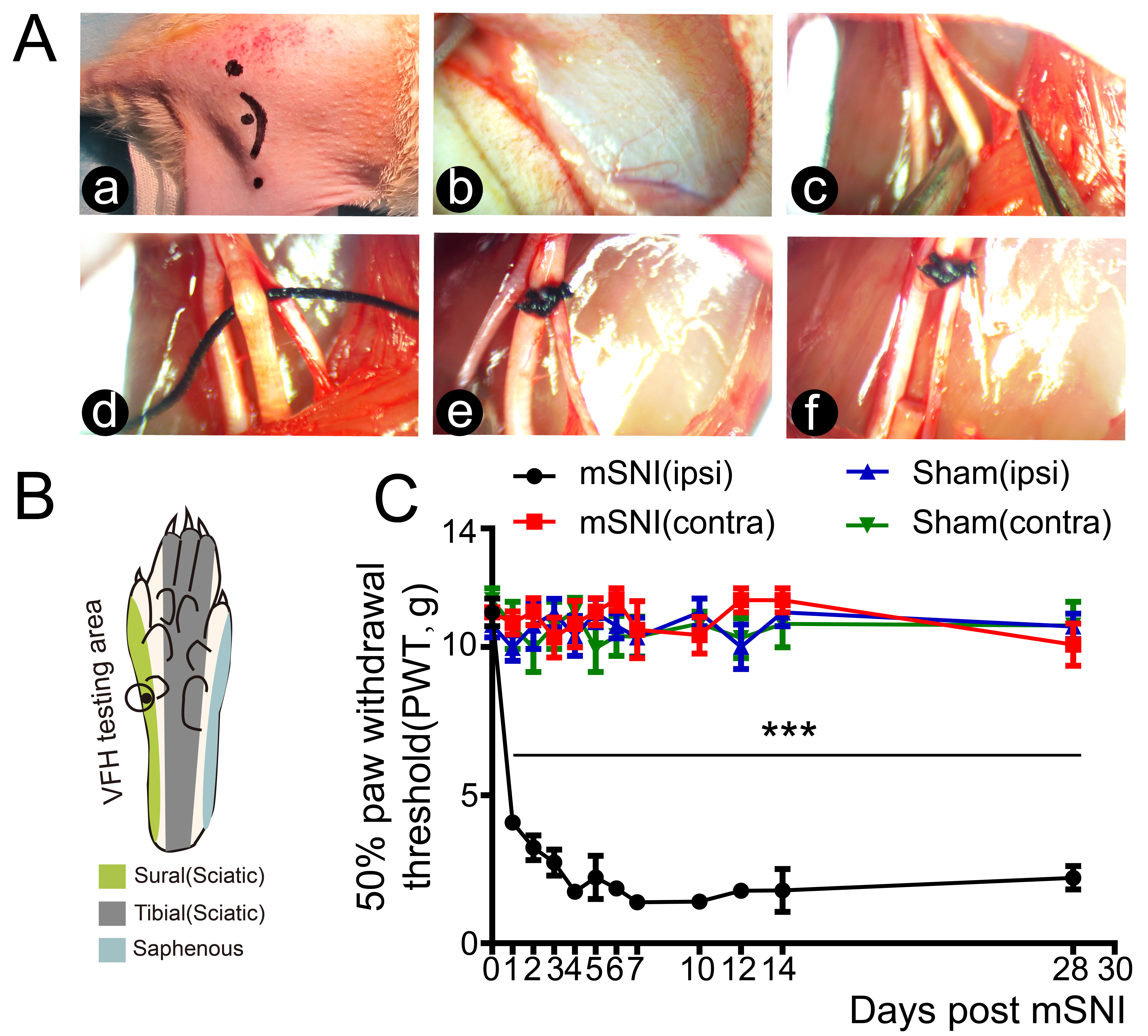

Supplement: Supplementary file 1 — Figure S1. The development of chronic mechanical allodynia after adult rat mSNIs. (A) Schematic illustration of surgical procedures for mSNIs, i.e., tibial nerve injuries, on the right hindlimbs. (B) VFH behavioral testing for mechanical allodynia on the glabrous sural skin areas, i.e., the lateral plantar surfaces of the hindpaws. (C) Temporal dynamics of 50% PWTs (g) for both ipsilateral (ipsi) and contralateral (contra) hindpaws before and after mSNIs or sham surgeries (n = 6/group). ***P < 0.001; mSNIs versus sham surgeries for ipsilateral hindpaws. mSNI: modified spared nerve injury; PWT: paw withdrawal threshold; VFH, von Frey hair. (JPEG 2492 kb) [file 12974_2018_1115_MOESM1_ESM.jpg]

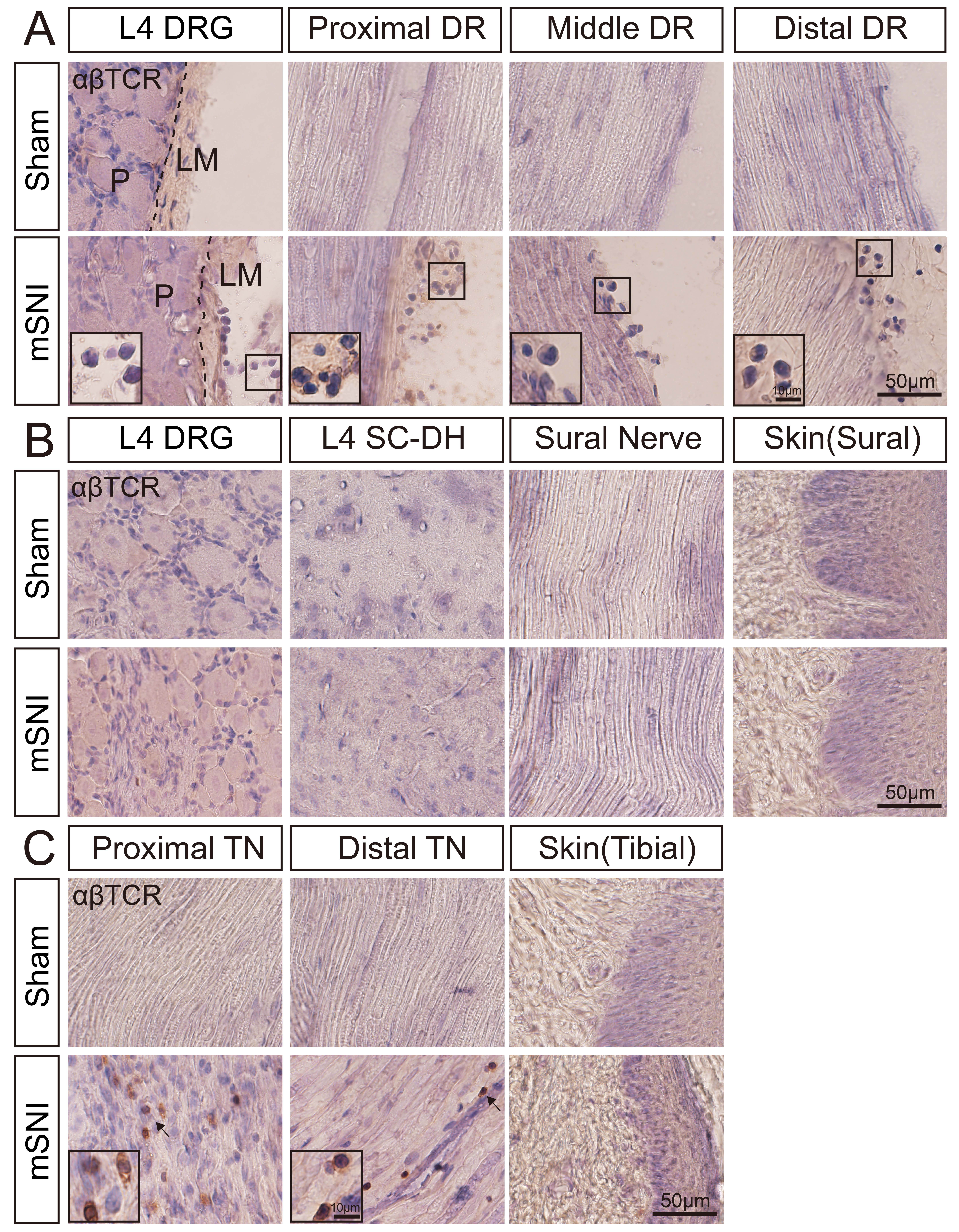

Supplement: Supplementary file 2 — Figure S2. Mapping of αβ T cells along the somatosensory pathways 7 days after adult rat mSNIs or sham surgeries with chromogenic IHC for αβTCR. (A) The infiltration of αβ T cells across the whole course of the L4 DR leptomeninges 7 days after mSNIs and sham operations. (B) The infiltration of αβ T cells in the cell-body-rich areas of L4 DRGs, L4 SC-DHs, the sural nerves, and the hindpaw glabrous sural skins 7 days after mSNIs and sham operations. (C) The infiltration of αβ T cells in the proximal and distal stumps of the injured tibial nerves and the hindpaw glabrous tibial skins 7 days after mSNIs and sham operations. DR: dorsal root; DRG: dorsal root ganglion; LM: leptomeninge; mSNI: modified spared nerve injury; P: parenchyma; SC-DH: spinal cord dorsal horn; TN: tibial nerve. (JPEG 4787 kb) [file 12974_2018_1115_MOESM2_ESM.jpg]

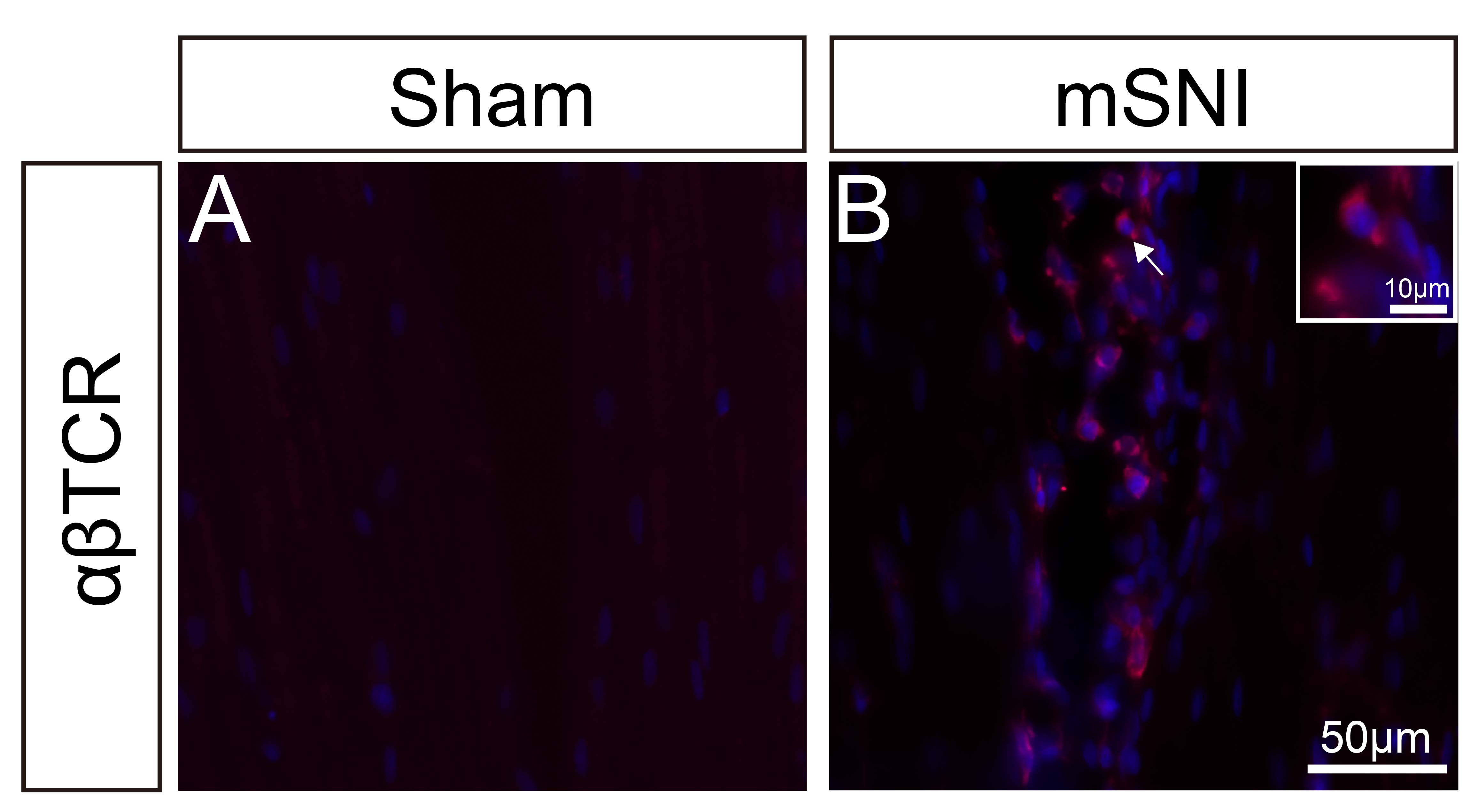

Supplement: Supplementary file 3 — Figure S3. AβTCR+ T cells in the pia maters perforating in the parenchyma of the proximal L4 DRs 7 days after mSNIs (B) and sham surgeries (A). n = 5/group. mSNI: modified spared nerve injury. (JPEG 246 kb) [file 12974_2018_1115_MOESM3_ESM.jpg]

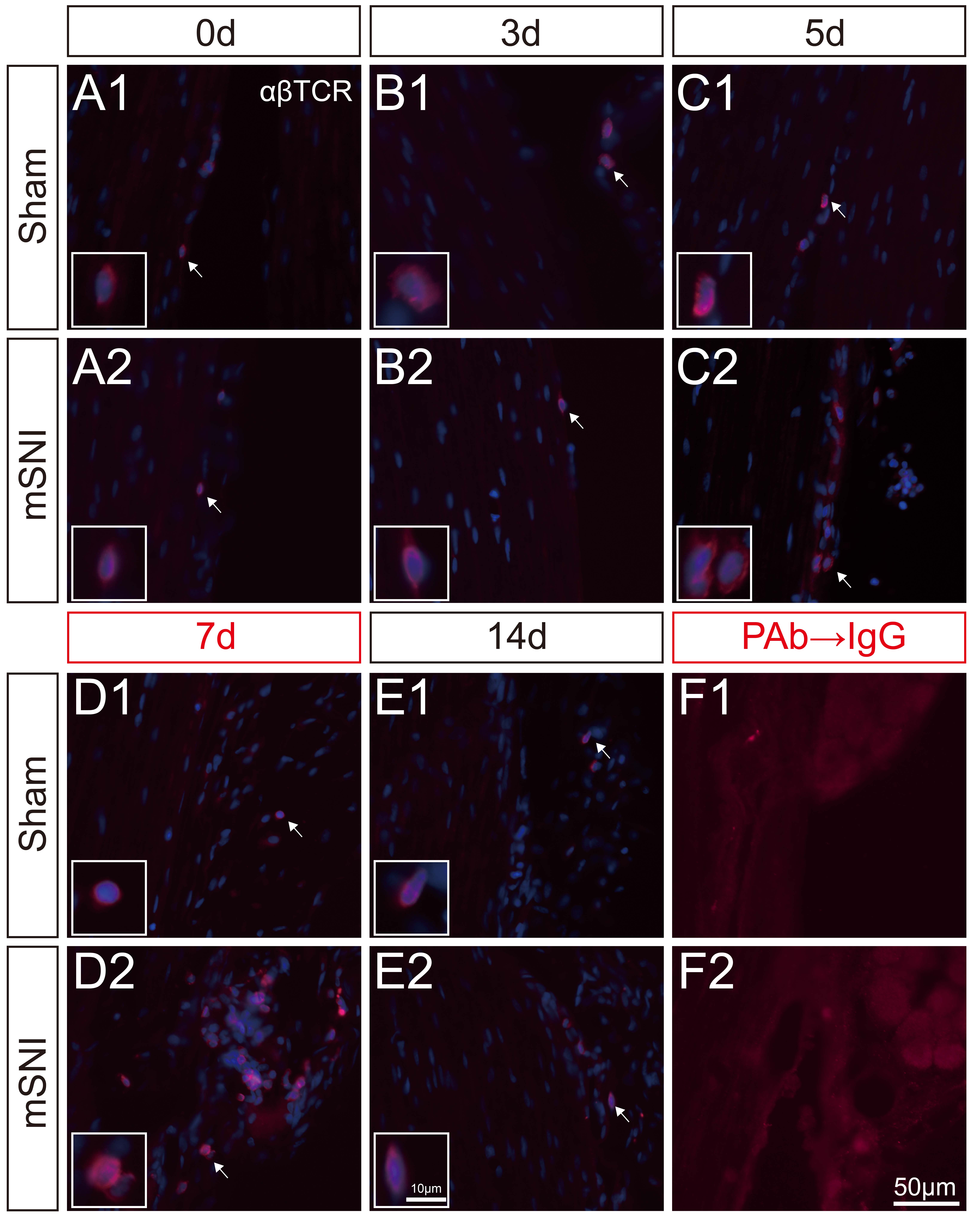

Supplement: Supplementary file 4 — Figure S4. The temporal dynamics of αβTCR+ T cell infiltration into the leptomeninges covering the proximal L4 DRs at the DR portions of the subarachnoid angles after mSNIs and sham operations (n = 5/group for each time point). Images in the white boxes show high magnified views of positive cells in the respective images of low magnification. (F1-F2) The corresponding staining control for D1 and D2 images by substituting primary antibodies with the corresponding isotype control IgGs. mSNI: modified spared nerve injury. (JPEG 1087 kb) [file 12974_2018_1115_MOESM4_ESM.jpg]

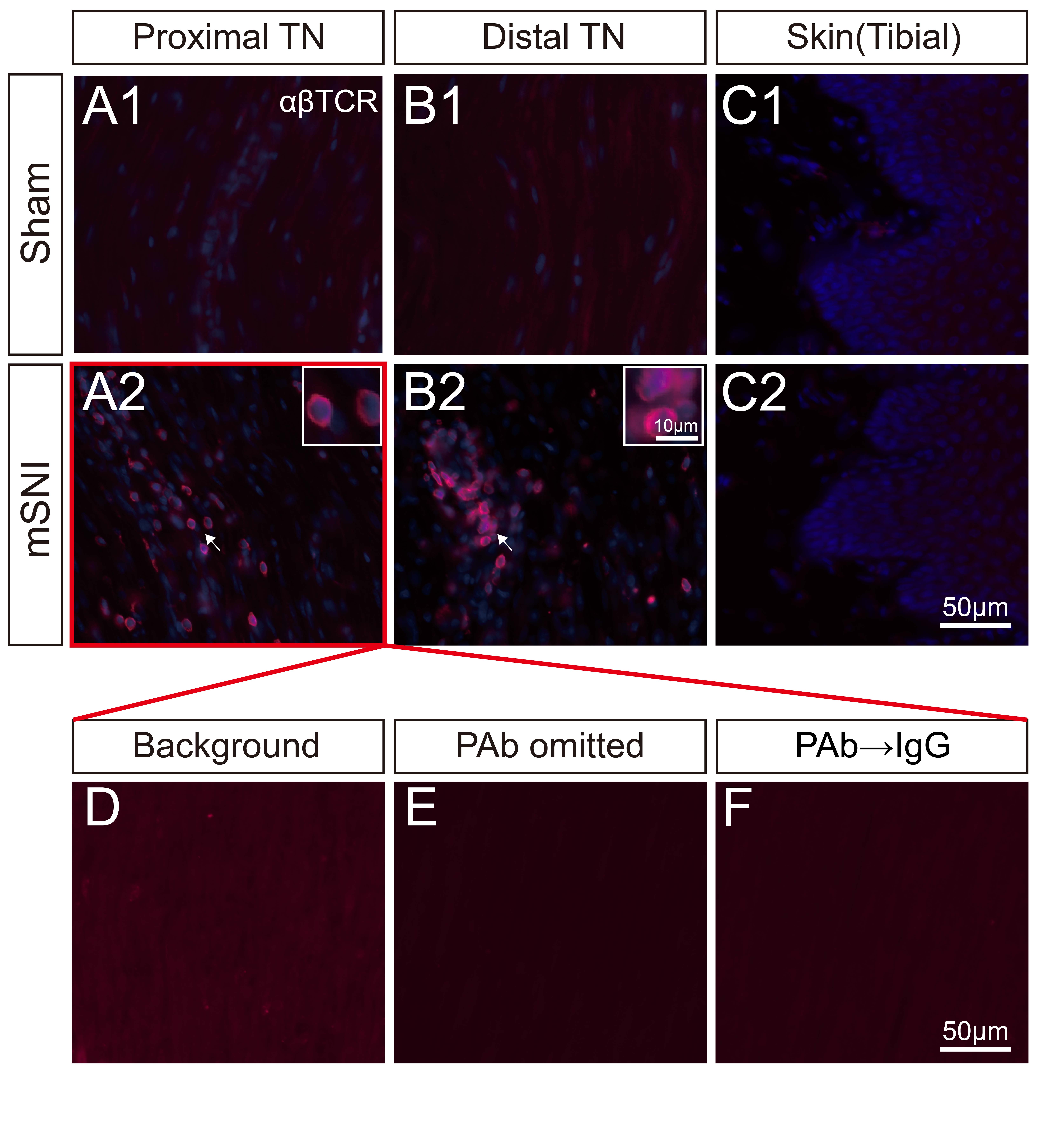

Supplement: Supplementary file 5 — Figure S5. The infiltration of αβ T cells in the proximal and distal stumps of the injured tibial nerves and the hindpaw glabrous tibial skins 7 days after mSNIs and sham operations. n = 5/group. (D–F) The corresponding staining controls for A2 image. mSNI: modified spared nerve injury; TN: tibial nerve. (JPEG 952 kb) [file 12974_2018_1115_MOESM5_ESM.jpg]

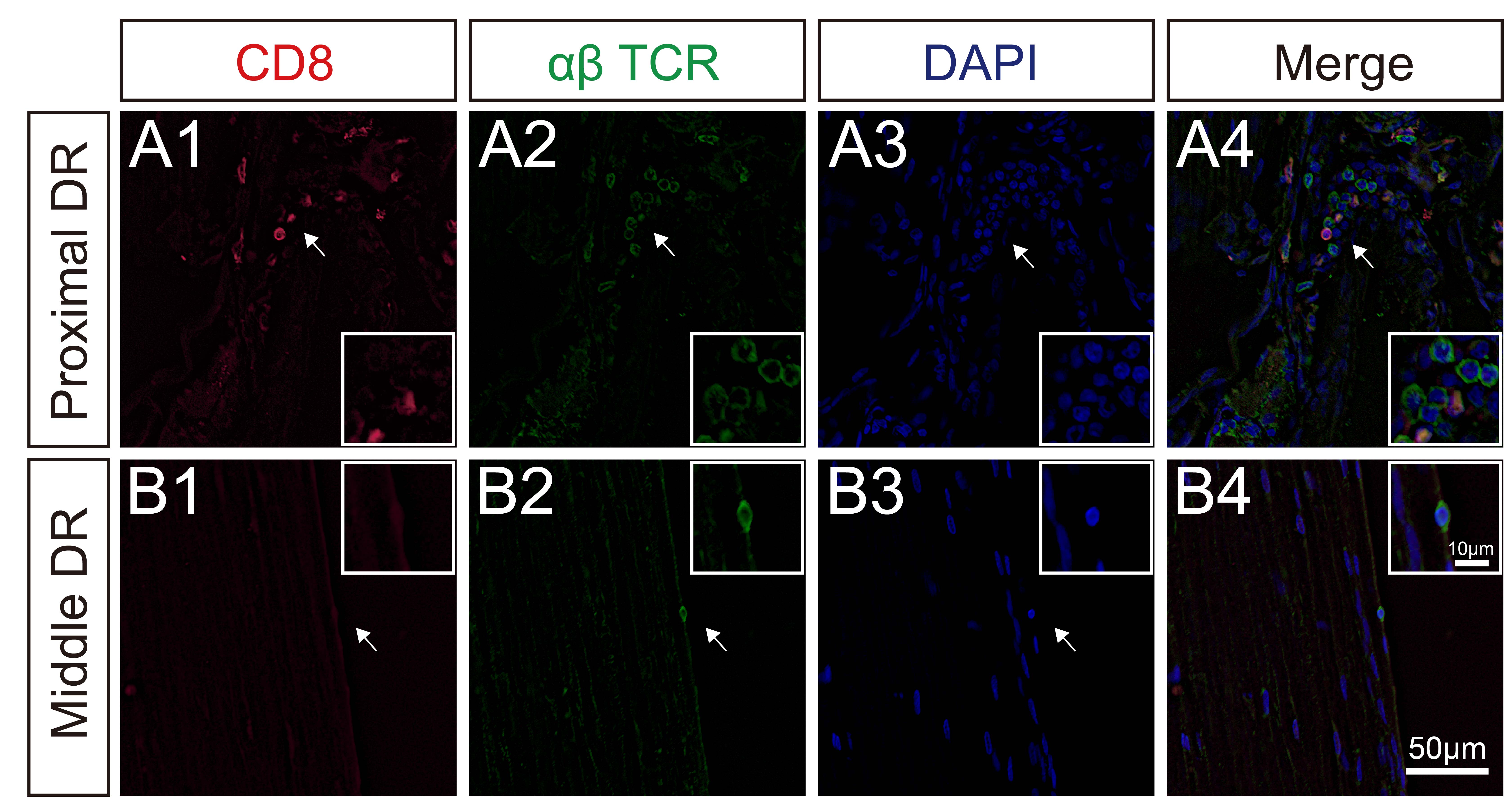

Supplement: Supplementary file 6 — Figure S6. The molecular identity of αβ T cells infiltrating into the lumbar DR leptomeninges 7 days after mSNIs. CD8 and αβTCR double staining of the L4 DR leptomeninges at the proximal DR (A1–A4) and the middle DR (B1–B4). DR: dorsal root. (JPEG 1050 kb) [file 12974_2018_1115_MOESM6_ESM.jpg]

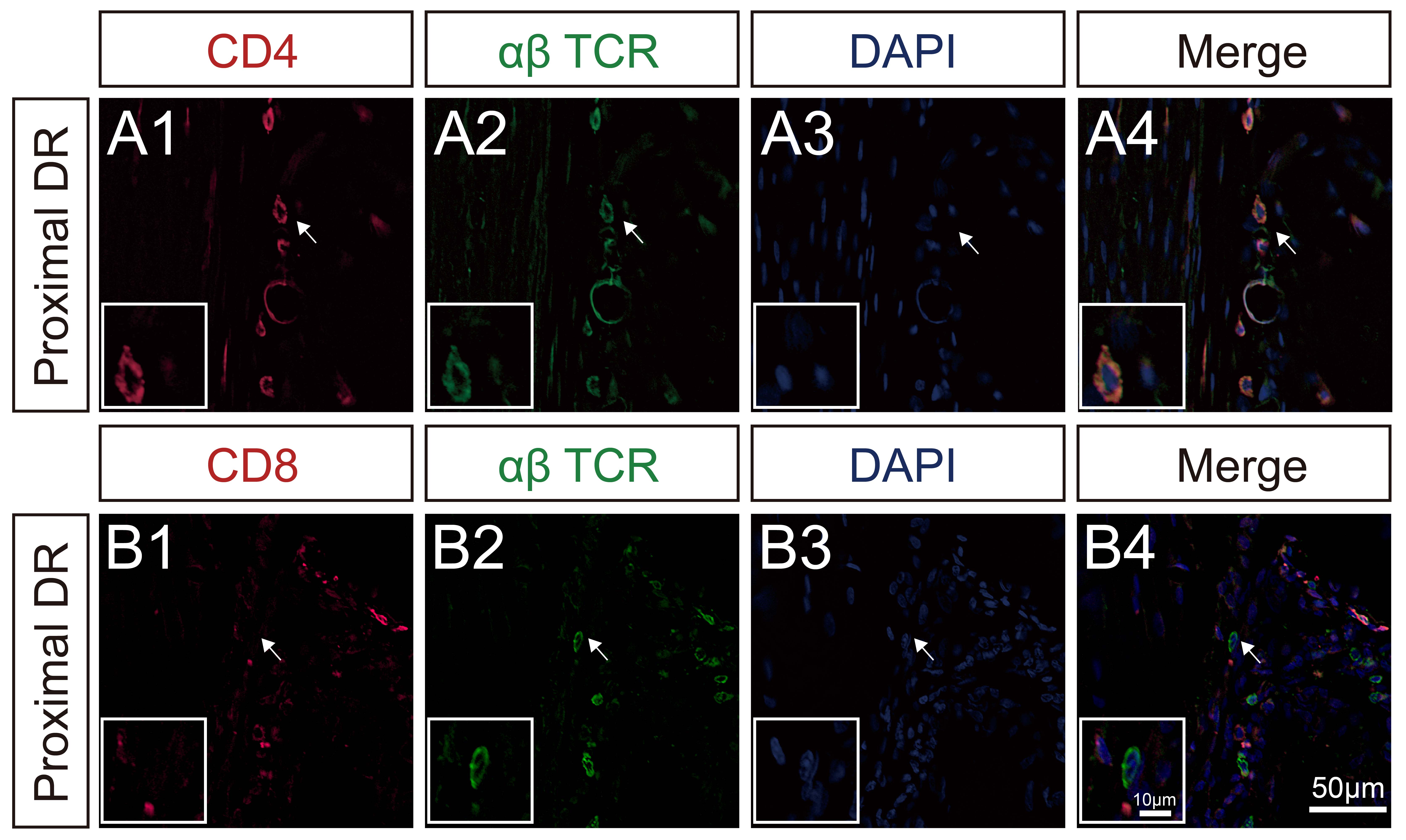

Supplement: Supplementary file 7 — Figure S7. The molecular identity of αβ T cells infiltrating into the lumbar DR leptomeninges 5 days after mSNIs. CD4 (A1–A4) or CD8 (B1–B4) and αβTCR double staining of the L4 DR leptomeninges at the proximal DR. DR: dorsal root. (JPEG 988 kb) [file 12974_2018_1115_MOESM7_ESM.jpg]

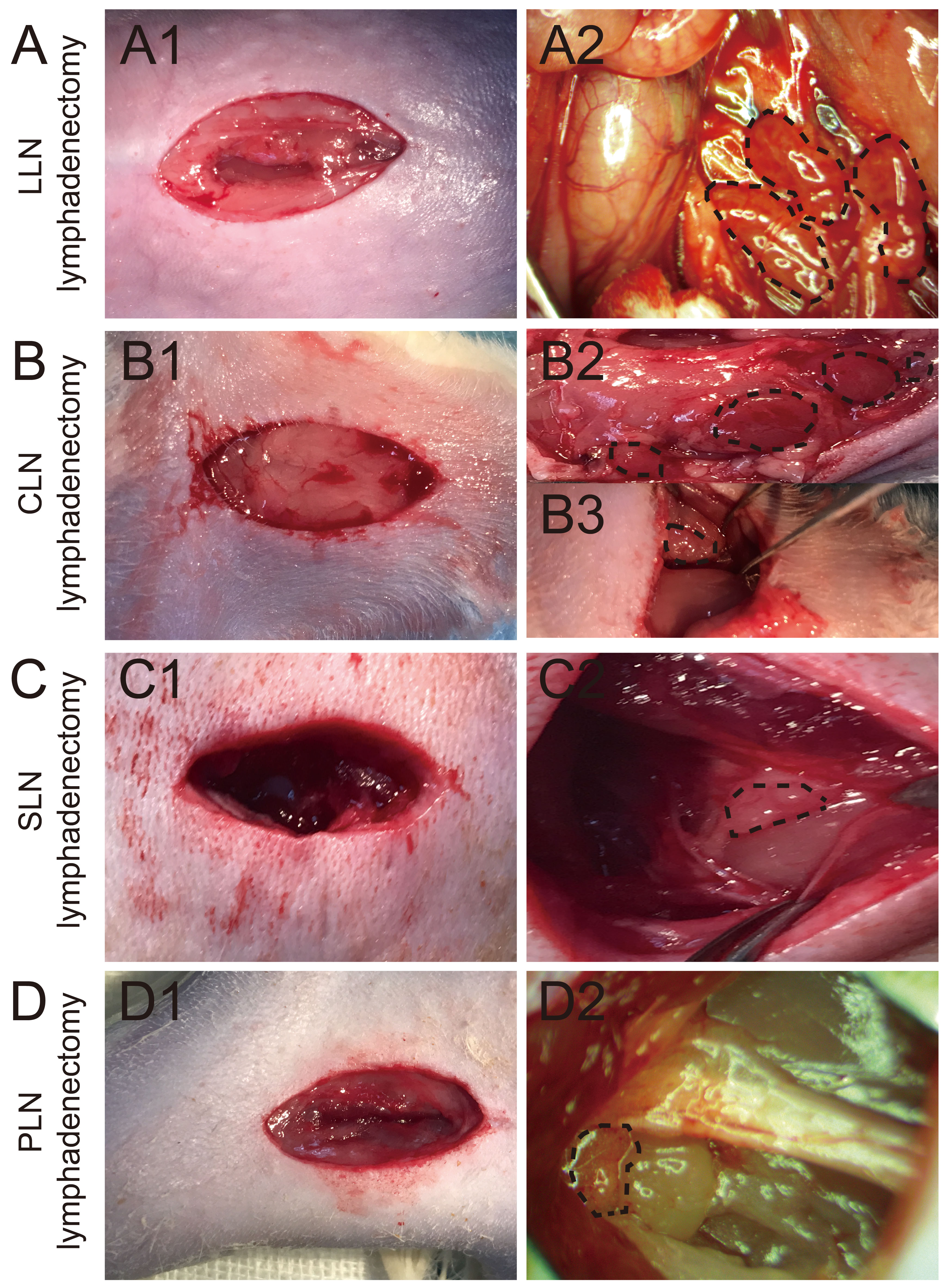

Supplement: Supplementary file 8 — Figure S8. Schematic illustration of surgical procedures for prior lymphadenectomies to LLNs (A), CLNs (B), SLNs (C), and PLNs (D) 7 days before mSNIs on the right hindlimbs. Left panel: skin incisions; right panel: the corresponding local lymph nodes (dashed circles). CLN, cervical lymph node; LLN, lumbar lymph node; PLN, popliteal lymph node; SLN, sciatic lymph node. (JPEG 2191 kb) [file 12974_2018_1115_MOESM8_ESM.jpg]

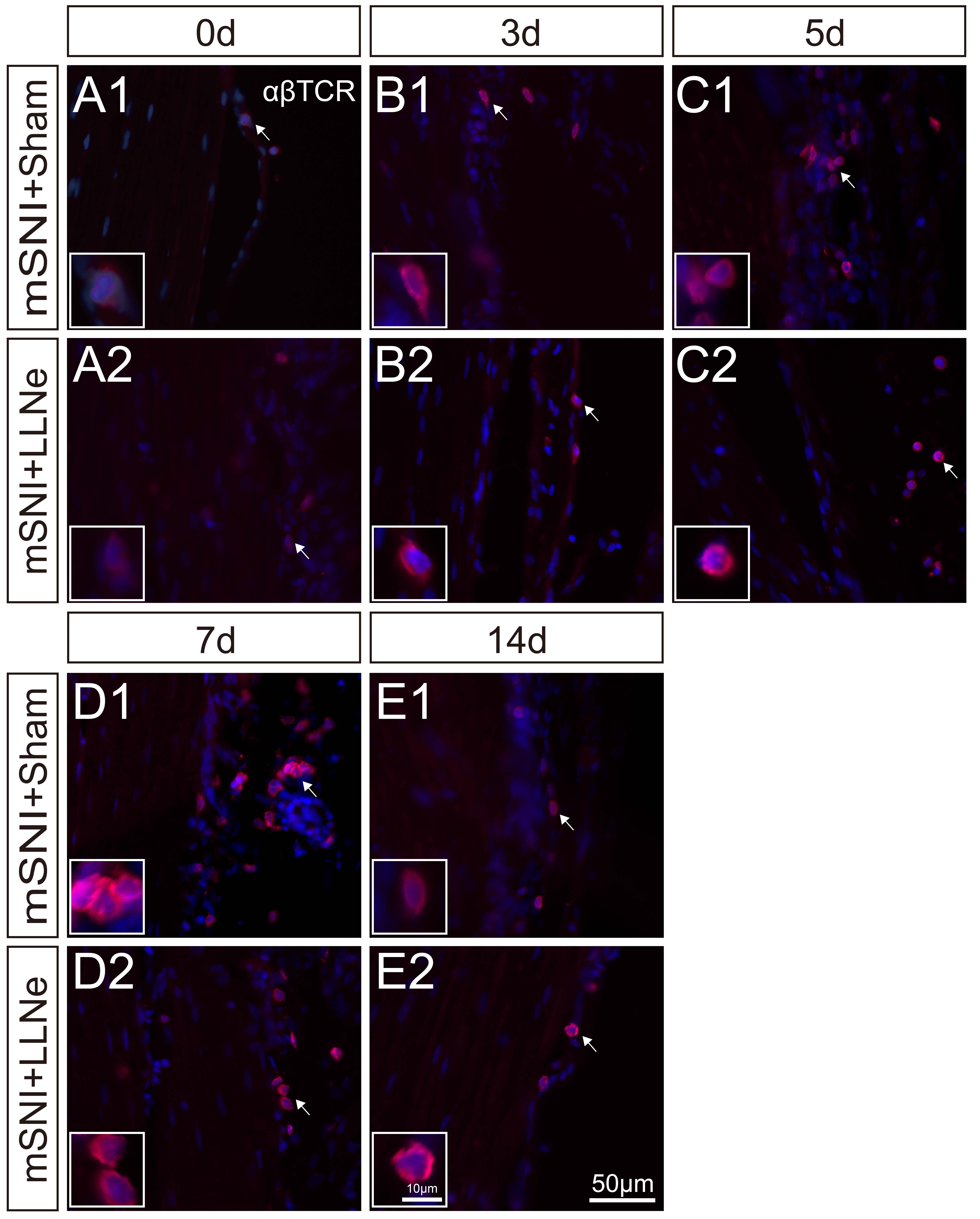

Supplement: Supplementary file 9 — Figure S9. Temporal dynamics of αβTCR+ T cell entry into the leptomeninges covering the proximal L4 DRs at the DR portions of the subarachnoid angles before and after mSNIs in prior lymphadenectomized or sham-operated animals to LLNs (n = 5/group). LLN: lumbar lymph node; mSNI: modified spared nerve injury. (JPEG 990 kb) [file 12974_2018_1115_MOESM9_ESM.jpg]

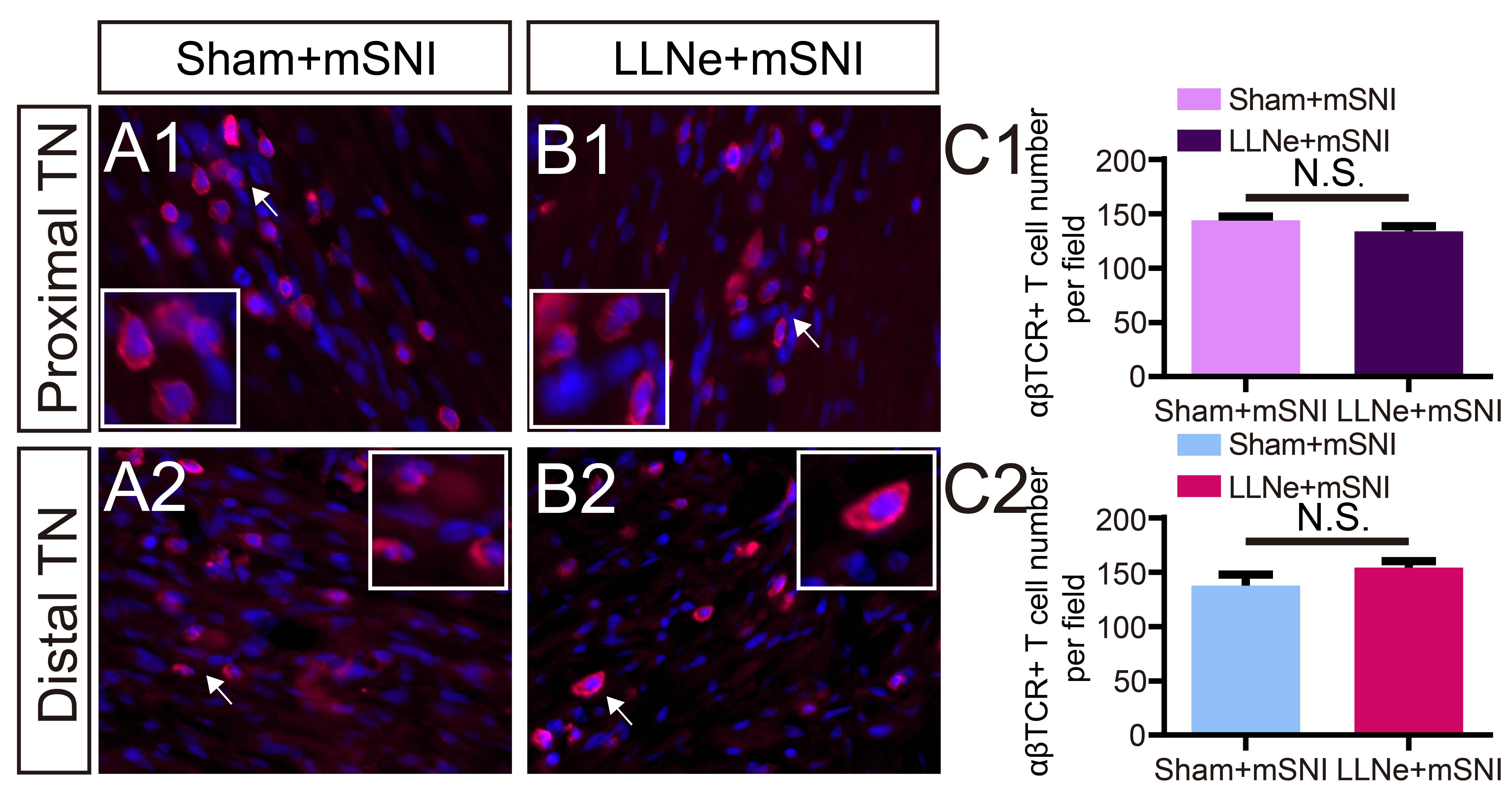

Supplement: Supplementary file 10 — Figure S10. Representative images and quantitative presentations for the numbers of αβ T cells in the proximal (A1–C1) or distal (A2–C2) stumps of the injured tibial nerves 7 days after mSNIs in prior lymphadenectomized or sham-operated animals to LLNs (n = 5/group). N.S., no significance; lymphadenectomy versus sham-operation. LLN: lumbar lymph node; mSNI: modified spared nerve injury. (JPEG 628 kb) [file 12974_2018_1115_MOESM10_ESM.jpg]

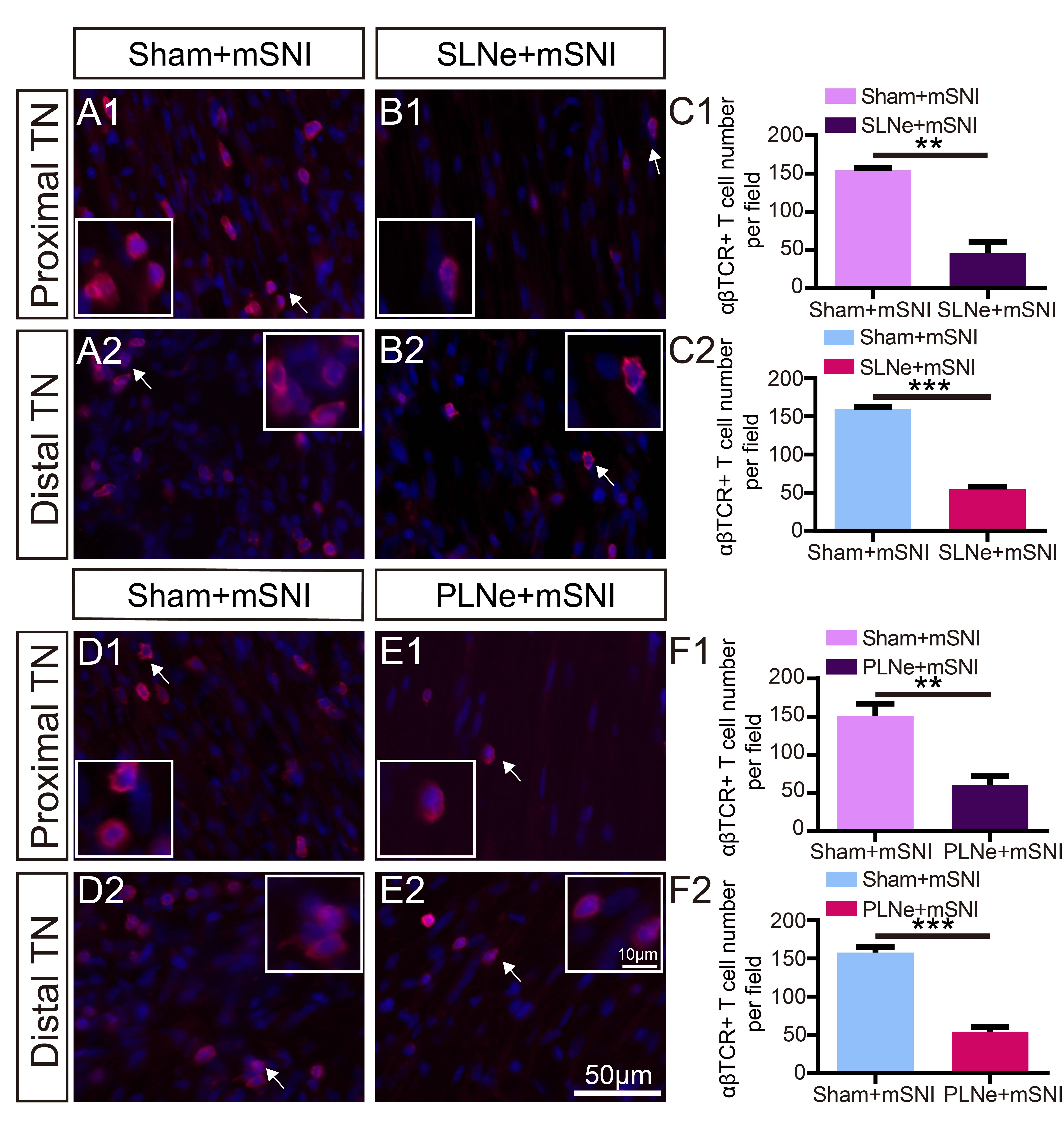

Supplement: Supplementary file 11 — Figure S11. Representative images and quantitative presentations for the numbers of αβ T cells in the proximal (A1–C1, D1–F1) or distal stumps (A2–C2, D2–F2) of the injured tibial nerves 7 days after mSNIs in prior lymphadenectomized or sham-operated animals to SLNs (A1–C1, A2–C2) or PLNs (D1–F1, D2–F2) (n = 5/group). **P < 0.01; *** P < 0.001; lymphadenectomy versus sham operation. SLN: sciatic lymph node; PLN: popliteal lymph node; mSNI: modified spared nerve injury. (JPEG 1098 kb) [file 12974_2018_1115_MOESM11_ESM.jpg]

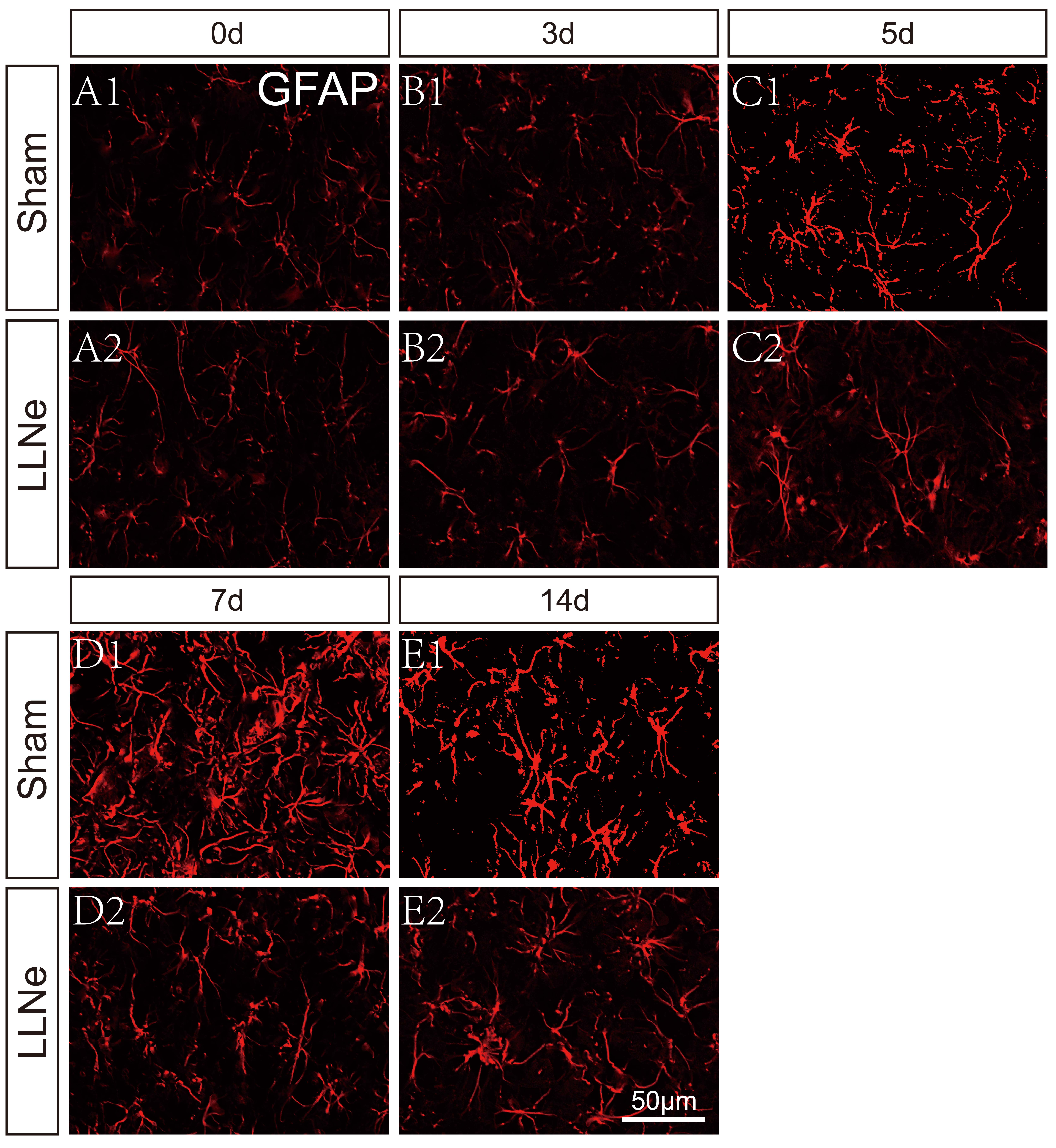

Supplement: Supplementary file 12 — Figure S12. Representative images for temporal dynamics of astrocyte activation in the sural projection areas of L4 SC-DHs before and after mSNIs in lymphadenectomized or sham-operated animals to LLNs (n = 5/group). LLN: lumbar lymph node; mSNI: modified spared nerve injury. (JPEG 2303 kb) [file 12974_2018_1115_MOESM12_ESM.jpg]

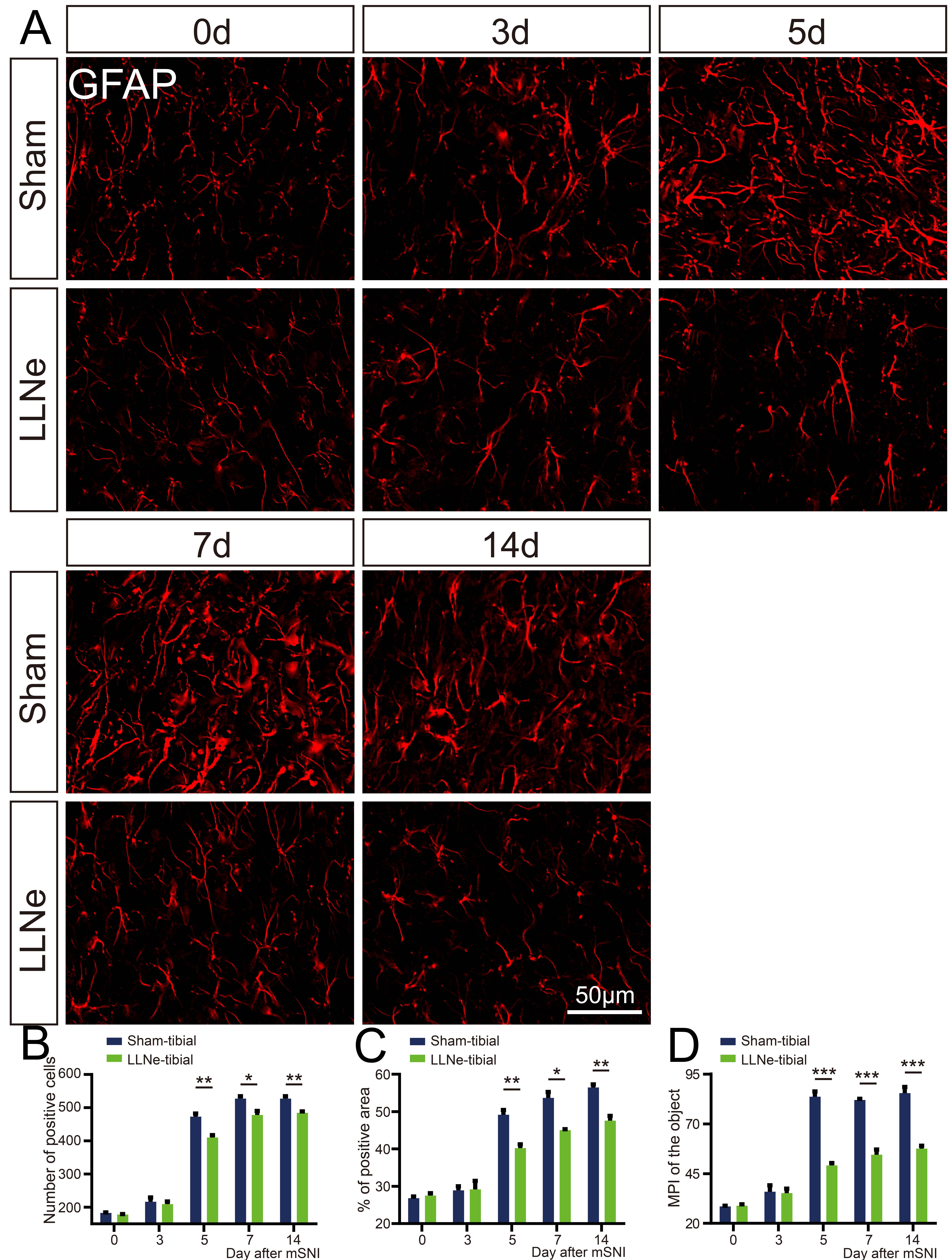

Supplement: Supplementary file 13 — Figure S13. Representative images (A) and quantitative presentations (C-D) for temporal dynamics of astrocyte activation in the tibial projection areas of L4 SC-DHs before and after mSNIs in lymphadenectomized or sham-operated animals to LLNs (n = 5/group). *P < 0.05; **P < 0.01; *** P < 0.001; lymphadenectomy versus sham operation. LLN: lumbar lymph node; mSNI: modified spared nerve injury. (JPEG 2624 kb) [file 12974_2018_1115_MOESM13_ESM.jpg]

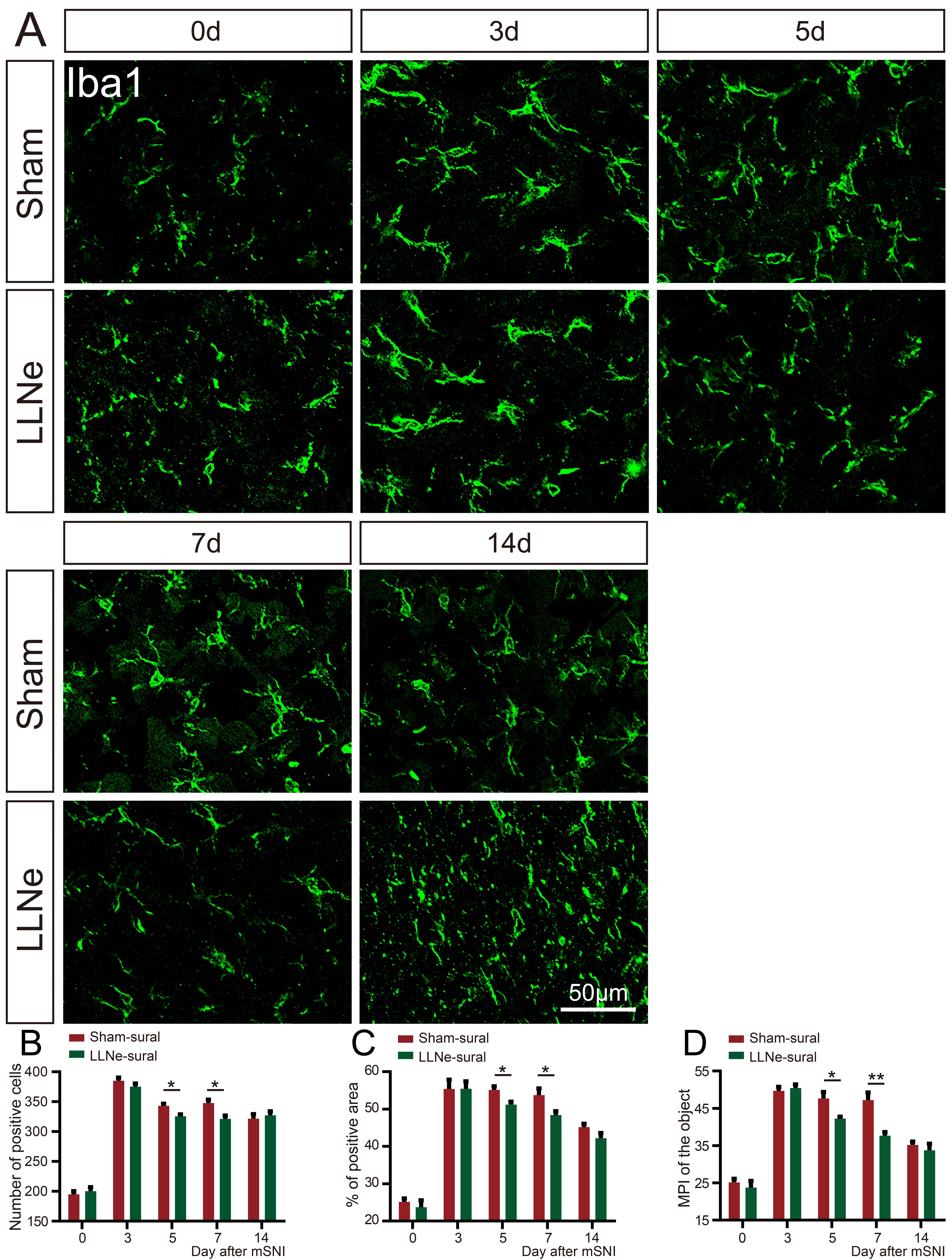

Supplement: Supplementary file 14 — Figure S14. Representative images (A) and quantitative presentations (C-D) for temporal dynamics of microglia activation in the sural projection areas of L4 SC-DHs before and after mSNIs in lymphadenectomized or sham-operated animals to LLNs (n = 5/group). *P < 0.05; **P < 0.01; lymphadenectomy versus sham operation. LLN: lumbar lymph node; mSNI: modified spared nerve injury. (JPEG 3931 kb) [file 12974_2018_1115_MOESM14_ESM.jpg]

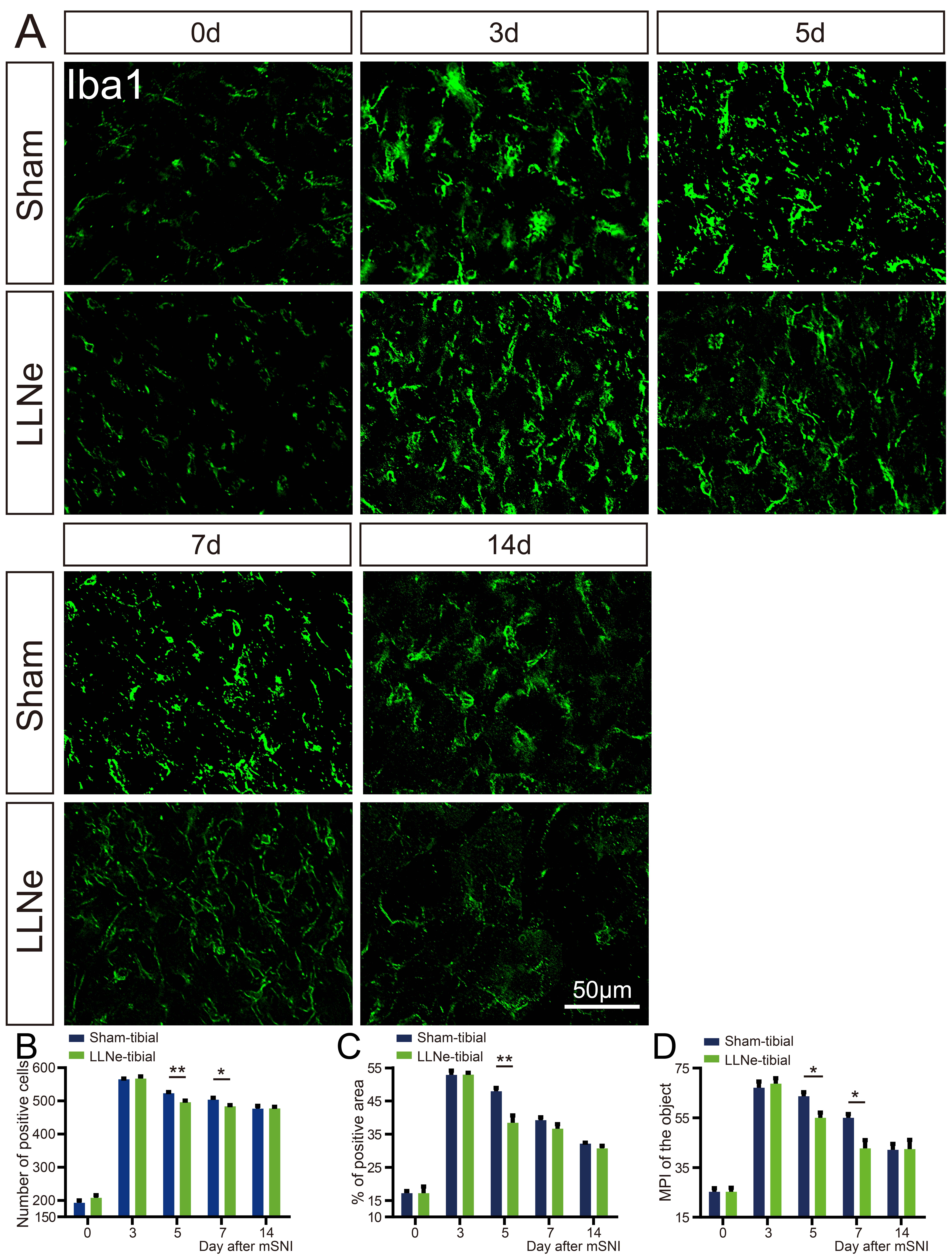

Supplement: Supplementary file 15 — Figure S15. Representative images (A) and quantitative presentations (C-D) for temporal dynamics of microglia activation in the tibial projection areas of L4 SC-DHs before and after mSNIs in lymphadenectomized or sham-operated animals to LLNs (n = 5/group). *P < 0.05; **P < 0.01; lymphadenectomy versus sham operation. LLN: lumbar lymph node; mSNI: modified spared nerve injury. (JPEG 3757 kb) [file 12974_2018_1115_MOESM15_ESM.jpg]
